# Genetically predicted triglyceride levels and endometriosis risk: Mendelian randomization with exploratory multi-omics analyses

**DOI:** 10.1371/journal.pone.0356100

**Published:** 2026-08-13

**Authors:** Yonghong Li, Jianan He, Yuanqing Zhang, Min Yang, Zhimao Jia, Yidan Fan, Shuyun Liu, Mengsha Qi

**Affiliations:** 1 Obstetrics and Gynecology, Wenjiang District People’s Hospital, Chengdu, Sichuan, China; 2 School of Clinical Medicine, Chengdu Medical College, Chengdu, Sichuan, China; 3 Library, Wenjiang District People’s Hospital, Chengdu, Sichuan, China; 4 School of Life Science and Technology, University of Electronic Science and Technology of China, Chengdu, Sichuan, China; Charite Universitatsmedizin Berlin, GERMANY

## Abstract

This study explored the genetic evidence linking triglyceride-related pathways and lipid-lowering drug targets with endometriosis risk and investigated the genetic association between TG levels and EM. Using genome-wide association study (GWAS) data, a two-sample Mendelian randomization (MR) analysis was conducted, estimating the association between genetically predicted TG levels and EM risk (OR = 1.1853, 95% CI: 1.070–1.313, *p* = 0.0011). Seven TG-lowering drug target genes (*SLC9A1, SCN3A, VEGFA, APOA1, TNF, ITGAV, BACE1*) were identified. Transcriptome analysis revealed that *SCN3A, BACE1,* and *APOA1* were significantly upregulated in EM patients compared to healthy controls. Enrichment pathway analysis showed that these genes are involved in lipid transport, localization, cell adhesion, and blood vessel development. *PPARA* was predicted to act as a transcription factor for five of these genes, while estrogen receptor 1 (ESR1) was predicted to regulate the majority of the others. Among the TG inhibitors examined, fibrate-related targets showed relatively broader target coverage in the present genetic analyses. These findings suggest that TG-related pathways and lipid-lowering drug targets, particularly fibrate-related targets, may be relevant to EM biology and warrant further experimental and clinical validation.

## Introduction

Endometriosis (EM) is a prevalent condition among women of reproductive age, affecting approximately 190 million women worldwide [1]. It is characterized by the presence of endometrial tissue (comprising glands and stroma) outside the uterine cavity, leading to symptoms such as dysmenorrhea, abnormal menstruation, dyspareunia, infertility, and more. Affecting approximately 10% of women [2], EM significantly impacts quality of life. These symptoms disrupt daily activities and reduce work efficiency, with pelvic pain and disease severity accounting for up to 60% of productivity loss.

EM is known for its estrogen dependence, but its exact underlying causes remain incompletely understood. Currently, laparoscopic surgery is considered the gold standard for diagnosing EM. However, due to its invasive nature, it may not be suitable for early diagnosis or monitoring the progression of the disease. To date, no peripheral blood or endometrial biomarkers have suggested sufficient accuracy in diagnosing EM. Given the chronic nature of EM, long-term pharmacological management is essential. While surgical intervention can provide symptomatic relief and remove existing lesions, it does not offer a permanent solution and carries significant costs. Ahmed M Soliman et al. found that the average annual cost of medical care for patients with endometriosis was more than three times that of non-endometriosis controls ($16,573 vs $4,733; *p <* 0.005) [3]. At the same time, the surgical recurrence rate was high. One systematic evaluation showed a persistence or recurrence rate of 22% at 2 years and 40%–50% at 5 years after surgery [4]. Therefore, the development and investigation of effective medications for long-term management and symptom control are important aspects of managing EM [5]. These medications can help alleviate pain and improve overall quality of life for individuals with EM. In summary, further exploration is needed to better understand the pathogenesis of EM, develop improved diagnostic methods, and establish effective disease control strategies. Given the unmet need for accurate diagnosis and effective treatment options for EM, exploring the potential of existing drugs to improve the condition of patients with EM may serve as a viable alternative. Considering the large population of individuals affected by EM, utilizing currently available medications may provide relief and help manage the symptoms associated with the condition.

Recent clinical research has shown a significant elevation in triglyceride levels among patients with EM compared to healthy women [6]. Meanwhile, the findings of Hongling Zhang et al. provided evidence consistent with a potential causal relationship between TG and increased risk of EM (OR = 1.112, 95% CI: 1.033–1.198, *p* = 5.03 × 10 ⁻ ³). They concluded that higher serum TG levels were associated with a greater risk of EM [7]. Additionally, several studies have established a positive correlation between triglyceride levels and the severity of EM [8]. Triglycerides (TG) play a crucial role in the structural composition of living organisms and actively engage in diverse signaling pathways, exerting varying degrees of influence on cellular functions. TG is implicated in processes such as inflammation, immunity, and steroid hormone metabolism, and their close association with the onset and progression of certain diseases has been well-documented. Nevertheless, the precise nature of the association between TG and EM remains elusive. Consequently, it is imperative to investigate the genetic association between TG and EM, thus fostering a deeper comprehension of the pathogenesis of this condition and subsequently formulating a theoretical basis for its diagnosis and treatment.

Currently, the main triglyceride inhibitors encompass fibrates, ω-3 PUFA, and niacin medications.

Fibrates primarily target peroxisome proliferator-activated receptor-α (PPARA), leading to a reduction in triglyceride (TG) levels by 20% to 50% [9]. Fibrates function as agonists of PPARA, binding to and activating this nuclear receptor. PPARA exhibits predominant expression in tissues actively engaged in lipid metabolism, including the liver, adipose tissue, and skeletal muscle. Upon binding to PPARA, fibrates modulate the expression of genes associated with lipid and lipoprotein metabolism, leading to diverse physiological effects, such as heightened fatty acid oxidation, diminished triglyceride synthesis, and enhanced high-density lipoprotein cholesterol (HDL-C) production [10]. While fibrates primarily exert their action on PPARA, it is worth noting that PPARA itself possesses the ability to regulate the expression of numerous genes and influence diverse metabolic pathways involved in lipid and glucose metabolism. Therefore, despite fibrates having a singular primary target, their effects can transcend PPARA and indirectly engage multiple targets by means of downstream effects resulting from PPARA activation. For instance, fibrate-induced activation of PPARA upregulates the expression of genes involved in fatty acid transport, oxidation, and lipoprotein metabolism [11]. Consequently, this can instigate alterations in the activity of enzymes, transporters, and receptors implicated in these processes. Moreover, PPARA activation can modulate inflammatory and oxidative stress pathways, thereby additionally influencing gene expression and cellular processes [12]. In summary, while fibrates primarily target PPARA, they indirectly affect multiple targets by modulating gene expression and metabolic pathways associated with lipid and lipoprotein metabolism. Therefore, investigating the role of fibrates in EM can enhance our understanding of the relationship between PPARA and multiple metabolic targets, thereby providing a more scientific and rational basis for the use of fibrates.

Omega-3 polyunsaturated fatty acids (ω-3 PUFA) can lower triglyceride levels in the body through various mechanisms and by targeting multiple pathways. They can reduce TG levels by approximately 25% to 30% [13]. Regarding triglyceride synthesis, omega-3 fatty acids inhibit diacylglycerol acyltransferase (DGAT), an enzyme involved in the terminal stage of triglyceride synthesis. Through the reduction of DGAT activity, omega-3 fatty acids decrease triglyceride production in the liver [14]. Simultaneously, omega-3 fatty acids upregulate the activity of enzymes implicated in fatty acid oxidation, such as acetyl-CoA carboxylase 1 (ACC1) and fatty acid synthase (FAS), facilitating the breakdown (oxidation) of fatty acids in the liver. This reduces the availability of glycerol and fatty acids for triglyceride synthesis, consequently lowering triglyceride levels [15]. Additionally, omega-3 fatty acids can enhance the activity of lipoprotein lipase (LPL), an enzyme that facilitates the breakdown of TG into fatty acids and glycerol for utilization by tissues. This, in turn, increases the rate at which TG are cleared from the bloodstream [16]. Furthermore, omega-3 fatty acids can promote a favorable distribution of lipids, which includes reducing triglyceride levels, by modulating the expression of genes involved in lipid metabolism. Additionally, omega-3 fatty acids possess anti-inflammatory properties and contribute to cardiovascular health and neuroprotection [17]. Exploring the multi-target and multi-mechanism characteristics of omega-3 fatty acids may yield novel insights and treatment strategies for addressing the chronic inflammatory state, pain, and hormonal imbalance associated with EM.

Niacin is currently utilized in cases where fibrates and prescription-grade omega-3 fatty acids have been ineffective in managing TG, albeit its notable adverse effects. While the mechanism of action of niacin remains unclear, niacin appears to mitigate triglyceride and low-density lipoprotein cholesterol levels by modulating multiple targets involved in triglyceride metabolism within adipose tissue and the liver. Research indicates that niacin activates GPR109A, leading to the inhibition of lipolysis (the breakdown of stored fat) in adipose tissue. This, in turn, decreases the release of free fatty acids into the bloodstream, thereby aiding in the reduction of triglyceride synthesis in the liver [18]. Furthermore, niacin exerts a direct and non-competitive inhibitory effect on diacylglycerol acyltransferase 2 (DGAT2), a crucial enzyme involved in hepatic triglyceride synthesis.

Consequently, this inhibition leads to decreased triglyceride synthesis and reduced secretion of atherosclerotic lipoproteins from the liver [19]. Moreover, niacin medications can activate the PI3K/PKB pathway, leading to the inhibition of FAS activity in the liver. As a result, triglyceride synthesis is reduced [20]. Simultaneously, niacin plays a role in energy metabolism within the body, influencing cellular redox status and ATP production [21]. Abnormalities in cell metabolism are associated with the development of EM [22]. Investigating the mechanism of action of niacin in this context can offer valuable insights into the progression of the disease. Furthermore, niacin regulates immune cell function and modulates inflammatory responses [23], while promoting tissue repair [24] and exerting antioxidant effects [25]. EM frequently involves inflammatory responses to tissue damage and heightened oxidative stress. Investigating the mechanisms by which niacin promotes tissue repair and mitigates oxidative stress may contribute to the development of novel treatment strategies for EM.

**Fig 1:**
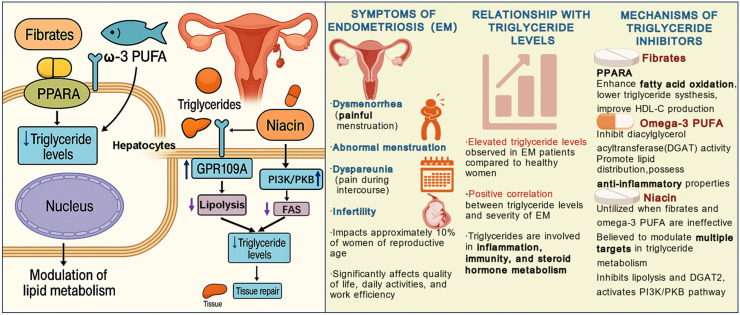
Detailed illustration of the introduction.

In conclusion, it is evident that a variety of triglyceride inhibitor drugs are currently available. However, selecting a suitable triglyceride inhibitor for EM patients remains a challenge. To provide genetic insights into the potential relevance of triglyceride-related drug targets in EM, our study employed the Mendelian randomization (MR) method to analyze the genetic associations between triglyceride inhibitor-related targets and EM risk. Our analysis aimed to evaluate the genetic evidence supporting the potential relevance of triglyceride-related targets in EM. Fig 1 provides a detailed overview of the study rationale.

## Methods

### Research design

Firstly, we utilized a two-sample univariate MR analysis to estimate the genetically predicted association of TG on EM. This analysis helped evaluate the genetic association between TG and EM and assess whether the findings were compatible with a potential causal effect.

Secondly, we conducted a search in the drug-gene interaction database to identify target genes associated with triglyceride drugs. By leveraging this database and referring to previous studies on druggable genes, we aimed to characterize specific genes that are targeted by triglyceride drugs and potentially play a role in the development or treatment of EM.

Thirdly, we employed the genetic variants that are mediated by the target genes and associated with TG to serve as proxies for TG-lowering drug targets. Using a two-sample MR approach, we investigated the association between genetic proxies for TG-lowering drug targets and EM risk by analyzing data from two Genome-Wide Association Studies (GWAS).

Fourthly, we conducted colocalization analysis to examine whether the association between TG and EM was driven by the same genomic locus. This analysis could help determine if there was shared genetic regulation underlying both traits.

Fifthly, we explored the protein interaction relationships among the selected genes and examined their enrichment functions. Additionally, we evaluated the significance and importance of the identified genes in the context of EM.

**Fig 2:**
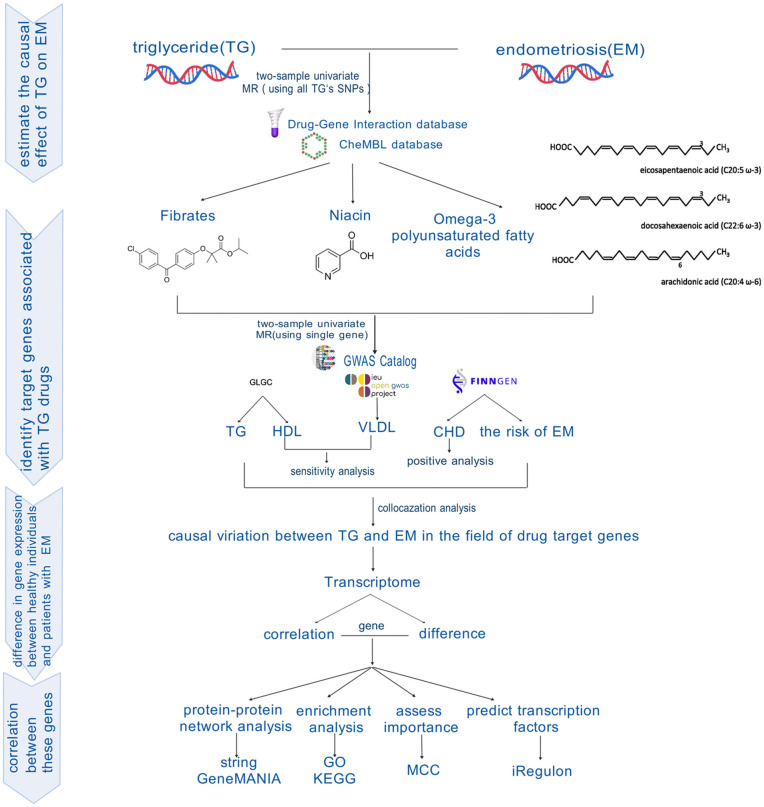
Overview of the study design and analytical process.

Finally, we searched for transcription factors (TFs) that regulated these genes, aiming to identify the regulatory mechanisms that influenced the expression and function of the identified genes in relation to EM. The overall study design and analysis procedure are displayed in Fig 2. The graphical summary is created by BioGDP [26].

### Genome-wide association study data sources and approval

Based on the information provided, the data used in the study were obtained from public databases and specific GWAS consortia. The sources of the GWAS data used in the analysis are as follows:

TG and high-density lipoprotein (HDL) data: The data were obtained from The Global Lipids Genetics Consortium (GLGC). This consortium conducts large-scale genetic studies to unravel the genetic architecture of lipid traits. The GLGC study included 188,577 participants, predominantly of European ancestry, consistent with the majority of its constituent studies. Plasma lipid concentrations were measured after a minimum fasting period of 8 hours. Estimates were adjusted for age, age squared, sex, and population stratification, with exclusion of participants known to be taking lipid-lowering medications. The selected genetic variants collectively explained 10–14% of the total trait variance. For individual variant association estimates, additive genetic models were fitted using inverse-normal transformed trait linear regression. For pooled effect estimates, weighted meta-analysis was performed using Stouffer’s method. In the European-specific analysis (approximately 1.3 million individuals, accounting for 80% of the total sample), the identified lipid-associated signals constituted 76% of all signals. The large sample size ensures that the effect estimates in the European population are relatively stable, and the overall results exhibit high consistency. HDL cholesterol was standardized, with the genetically predicted association reflecting the effect of a 1 standard deviation increase in HDL on the outcome. Triglycerides are log-transformed due to skewed distribution, with the genetically predicted association interpreted as the effect of doubling the triglyceride level [27].

Very low-density lipoprotein (VLDL) data: The data were sourced from the IEU OpenGWAS project. The IEU OpenGWAS project is a platform that provides access to a diverse array of GWAS datasets for different traits. VLDL levels were analyzed as a continuous variable, typically reported in mmol/L (standard unit in European GWAS consortia). Sample size: 115,082 participants.

EM and coronary heart disease (CHD) data: The data for these conditions were obtained from the FinnGen study. FinnGen is a research project in genomics and personalized medicine that aims to understand the genetic basis of diseases in the Finnish population. The FinnGen database includes 377,277 participants. In the FinnGen database, disease diagnoses are primarily derived from the Finnish National Health Registries and electronic health records (EHRs), encompassing inpatient and outpatient diagnoses, medication prescriptions, laboratory test results, and medical procedure documentation. All disease information is standardized using International Classification of Diseases (ICD) codes and cross-validated across multiple data sources to ensure accuracy. For specific conditions such as cancer, diagnoses are further validated through disease-specific registries (e.g., cancer registries) and pathological confirmation. Rare diseases are additionally corroborated using genomic data to enhance diagnostic precision [28].

It is important to note that the use of these data was approved by the respective institutional review boards, ensuring ethical considerations and participant privacy. Informed consent was obtained from all participants involved in the original GWAS studies.

See Table 1 for detailed information on data sources.

**Table 1:** Details of the genome-wide association studies used in the present study.

	Trait	Abbreviation	Database	Population	Sample size
Exposure	triglycerides	TG	Global Lipids Genetics Consortium Results	European	1.32 million
	high-density lipoprotein cholesterol	HDL-C	Global Lipids Genetics Consortium Results	European	1.32 million
	very-low-density lipoprotein	VLDL	IEU OpenGWAS project	European	115,082
Outcome	coronary heart disease	CHD	FinnGen	European	377,277
	endometriosis	EM	FinnGen	European	377,277

### Mendelian randomization and sensitivity analysis

#### Genetically predicted association between triglyceride and endometriosis.

Single nucleotide polymorphisms (SNPs) that meet the genome-wide significance level *(p* < 5 × 10^−8^) were chosen as instrumental variables (IVs). Independent genetic variants were identified using a cutoff of linkage disequilibrium (LD) values (threshold set to r2 < 0.001, kb = 10,000) to ensure the independence of the IVs [29]. Proxy SNPs were not utilized in this study. SNPs lacking necessary statistical information were directly removed.

The inverse variance weighting (IVW) method was utilized as the primary statistical approach. In the case of a single SNP, the genetically predicted association was estimated using the Wald ratio test. Weighted median, Simple mode, and Weighted mode methods were employed to assess model robustness. MR-Egger can identify horizontal pleiotropy in models, although it has limited statistical power [30]. Therefore, in cases where MR-Egger yielded significant results, we investigated the included SNPs using PhenoScanner, a comprehensive genotype-to-phenotype database [31]. Additionally, to exclude SNPs that may influence EM through other phenotypes, we employed the leave-one-out method to assess whether a single SNP exerted a disproportionate impact on the overall MR estimate [32]. To mitigate the possibility of reverse causation, we conducted an additional analysis to assess whether EM might influence triglyceride levels.

#### Genetic instruments for triglycerides drugs.

The primary triglyceride-lowering drugs comprise fibrates, ω-3 PUFA, and niacin. Genes encoding the target proteins of these triglyceride drugs were acquired from DGIDB and ChEMBL. All targets gathered from these two sources were verified using NCBI Gene to determine if they were human genes. Non-human genes were excluded. We excluded genes that were not predicted with 90% confidence to be active according to ChEMBL’s Target Predictions. We selected SNPs that exhibited a significant association with TG levels (*p* < 5 × 10^−8^) within a 100 Mb window surrounding the genomic region of the drug target [33]. To enhance the instrumental variable’s strength for each drug target gene, a less stringent threshold for independent clustering SNPs was employed (r^2^ < 0.3, kb = 100).

Restricting instrument selection to variants located within or near pharmacologically annotated target-gene regions was intended as a biologically informed strategy to strengthen the link between the selected variants and the predefined target-related pathways. Consistent with current methodological guidance, these regional instruments were treated as proxies for target-related genetic variation rather than as direct equivalents of pharmacological target inhibition or activation [34,35].

#### Positive control analysis.

Positive-control MR analyses were used to assess the biological plausibility of the pharmacogenetic instrument framework in a lipid-related disease context. Because triglyceride-lowering medications are used in the management of CHD [36], and genetic studies have provided evidence consistent with a potential causal relationship between TG and CHD risk [37], CHD was selected as a positive-control outcome [38]. Results from this analysis were interpreted only as a supportive consistency check for the target-region instrument framework, rather than as validation of gene-specific pharmacological effects or evidence of therapeutic efficacy for EM [34,35].

#### Sensitivity analysis.

In clinical studies, fibrates and niacins have been shown to reduce the risk of atherosclerosis by increasing concentrations of HDL-C and decreasing concentrations of TG in plasma [39, 40]. Consequently, HDL-C is utilized as a downstream biomarker of the effects of fibrates and niacins. ω-3 PUFA reduces the synthesis and secretion of VLDL particles and enhances the removal of TG from VLDL and chylomicron particles through the upregulation of LPL [41]. As a result, VLDL is employed as a downstream biomarker of the effects of ω-3 PUFA.

#### Colocalization analysis.

We employed colocalization analysis to investigate whether TG and EM share the same causal variant within the drug target gene region. This analysis aimed to provide additional validation regarding the robustness of including IV. In this study, we assessed the possible probabilities using the following hypotheses: H0 represents the scenario where the gene is not associated with TG or EM; H1 suggests that the gene is associated with TG but not with EM; H2 indicates that the gene is associated with EM but not with TG; H3 suggests that the gene is associated with both TG and EM, but the SNPs are independent of each other; and H4 proposes that the gene is associated with both TG and EM and that they share common SNPs.

#### Statistical software.

All analyses were conducted using R 4.2.2 statistical software. The ‘TwoSampleMR’ package was utilized for MR and drug target studies, while the ‘coloc’ package was employed for colocalization analysis.

### Transcriptome data sources and processing

#### Data sources.

The Gene Expression Omnibus (GEO) database is a publicly accessible repository that collects high-throughput gene expression data from research institutions worldwide. It is maintained by the National Center for Biotechnology Information (NCBI) [42]. The extensive collection of experimental data in the GEO database is widely utilized in various research fields, including gene regulation, disease mechanisms, and drug discovery.

Our objective was to investigate the expression patterns and potential regulatory relationships among genes involved in EM using the GEO database. To accomplish this, we downloaded six transcriptome datasets related to EM from the GEO database. The specific datasets we obtained for analysis are GSE23339 [43], GSE25628 [44], GSE58178 [45], GSE11691 [46], GSE7846 [47], and GSE7305 [48]. The details of each data set are shown in Table 2.

**Table 2:** Details of transcriptome inclusion datasets.

GEO ID	Author	Year	Patients	Control	Data type
GSE23339	Shannon M Hawkins	2011	10	9	RNA-seq
GSE25628	Stefania Crispi	2013	16	6	RNA-seq
GSE58178	D Monsivais	2014	6	6	RNA-seq
GSE11691	M Louise Hull	2008	9	9	RNA-seq
GSE7846	G Sha	2007	5	5	RNA-seq
GSE7305	Aniko Hever	2007	10	10	RNA-seq

#### Preprocessing.

In the statistical analysis conducted using R software, we performed preprocessing steps on the gene expression profiles obtained from the downloaded datasets. The following steps were carried out:

ID conversion: We performed ID conversion on the probes present in the expression profiles, converting them into corresponding gene names. This conversion enabled a more interpretable representation of the data.

Log2 transformation: To standardize part of the unstandardized dataset, we applied the log2 function. This transformation helped normalize the expression values, ensuring a more symmetric distribution and facilitating subsequent statistical analyses.

Extraction and merging of expression profiles: Next, we extracted the expression profiles of genes from each dataset separately and merged them. This consolidation allowed for a comprehensive analysis using a combined dataset.

Batch effect removal: Batch effects were defined as technical differences resulting from the processing and measurement of samples in different batches, unrelated to any biological variation in the experiment. To enhance the accuracy of statistical inference, we used the ‘sva’ package to remove batch effects and other unwanted noise from the merged data. This step helped eliminate potential confounding factors and reduced the impact of batch effects on the results [49].

Principal Component Analysis (PCA): Prior to and after removing the batch effect, we generated a PCA graph. This analysis helped assess the reduction of batch effects by visually comparing the distribution of samples. The aim was to minimize the influence of batch effects on the results and improve the robustness of subsequent analyses.

After completing these preprocessing steps, we obtained a total gene expression profile comprising 101 samples, including data from 56 EM patients and 45 healthy controls. This processed dataset serves as the basis for further research and analysis.

#### Statistical analysis.

To explore the expression patterns and mutual regulatory relationships of genes in the EM and control groups, we employed various R packages for visualization and analysis. The specific steps are as follows:

Histograms of gene expression: We utilized the ‘reshape2’ package and the ‘ggpubr’ package to create histograms comparing the gene expression levels between EM patients and healthy controls. This visualization helped identify any differences in gene expression patterns between the two groups.

Spearman correlation test: To assess the correlation of gene expression levels within the EM patient and healthy control groups separately, we employed the ‘corrplot’ package and performed Spearman correlation tests [50]. This analysis allowed us to evaluate the strength and direction of the relationships between gene expression levels. The results of these tests were visualized using the ‘ggplot2’ package.

Visualization of gene positions and genome structure: We utilized the ‘RCircos’ package to visualize the relationship between the positions of the eight genes on the chromosome and the genome structure. This visualization approach provides insights into the spatial organization and arrangement of these genes within the genome [51].

By implementing these steps, we can gain a better understanding of the expression patterns, correlations, and genomic relationships of the genes involved in EM and control groups. The visualizations generated through these analyses facilitate the interpretation and communication of the results.

### Gene association analysis

To explore the direct correlation between genes, the drug targets identified through MR analysis were input into the STRING database to establish a protein-protein interaction (PPI) network. This network helped uncover the interactions among the genes of interest.

In addition, a PPI network was also constructed in GeneMANIA to further examine the genes with significant indirect correlations and the functional roles they play. GeneMANIA provided insights into gene functions and interactions. The complementary nature of STRING and GeneMANIA has been suggested in several studies, with the former providing direct interactions and the latter extending indirect associations to improve the comprehensiveness and reliability of network-based functional annotation. STRING was used to initially construct the PPI network and identify core genes. GeneMANIA was further validated through enrichment analysis of the functional modules, which was cross-checked with the STRING results to reduce false positives [52].

To gain more insights from the constructed PPI networks, Gene Ontology (GO) and Kyoto Encyclopedia of Genes and Genomes (KEGG) enrichment pathway analyses were performed. The thresholds for significance were set as false discovery rate (FDR) < 0.05 and strength > 0.01. These analyses helped identify the biological processes and pathways associated with the genes in the network. The threshold of > 0.01 for interaction strength in STRING was selected based on the database’s recommendations and established practices in network analysis [53]. In addition, a threshold of strength >0.01 preserves weak but potentially biologically significant associations while avoiding excessive stringency leading to the omission of potential pathways. In our study, this threshold allowed us to broadly capture potential interactions among lipid metabolism and inflammation-related genes, which were further validated through functional enrichment and sensitivity analyses [54]. To evaluate the importance of the target genes in the network, maximal clique centrality (MCC) analysis was conducted using the CytoHubba plugin in Cytoscape software. This analysis assessed the centrality of the target genes, indicating their significance within the network.

Furthermore, the iRegulon plugin v1.3 [55] in Cytoscape software was employed to predict potential TFs associated with the key genes in the network. The enrichment score threshold was set to 3.0, area under the curve (AUC) threshold for the receiver operating characteristic (ROC) analysis was set to 0.03, ranking threshold was set to 5000, and the maximum FDR on motif similarity was set to 0.001. This analysis helped identify TFs that may regulate the expression of the key genes.

By performing these analyses and utilizing the mentioned tools and thresholds, this study aimed to gain insights into the gene interactions, functions, regulatory factors, and pathways associated with the identified drug targets.

## Results

### Genetically predicted association between triglycerides and endometriosis

Based on the analysis conducted, a total of 350 genome-wide significant SNPs associated with TG levels and 26 SNPs associated with EM were included as instrumental variables (IVs). The inverse-variance weighted (IVW) estimation was employed to assess the association between genetically predicted TG levels and EM risk.

**Fig 3:**
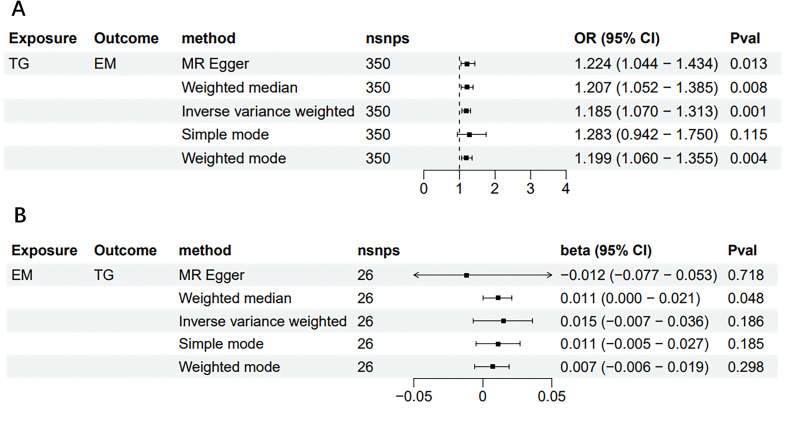
Genetically predicted association between TG and EM in univariable MR. (A) Genetically predicted associations of TG on EM; (B) Genetically predicted associations of EM on TG. The Fig shows the results of two data sets, A and B, involving different exposures and outcomes, demonstrating a comparison of several statistical methods. For group A, the exposure was TG and the outcome was EM. OR and *p* values show the effect estimates and statistical significance, respectively, for the different methods. ORs and their 95% confidence intervals (CIs) are represented as dots and lines in the Fig. For group B, the exposure was EM and the outcome was TG. Beta and *p* values show effect estimates and statistical significance, respectively. Nsnps represents the number of SNPs used for each method.

The results of the IVW estimation indicated that genetically predicted TG levels were significantly associated with EM risk (OR = 1.1853, 95% CI: 1.0704–1.3125, *p* = 0.0011). This suggests that higher genetically predicted TG levels are associated with an increased risk of EM (Fig 3A).

On the other hand, the analysis did not find a significant association between EM and TG levels (β = 0.0145, 95% CI: −0.0070 to 0.0361, *p* = 0.1862). This indicates that EM was not significantly associated with TG levels (Fig 3B).

Since the MR-Egger analysis yielded significant results (*p <* 0.05) in the TG-EM analysis, the leave-one-out method was utilized to test the significance of individual SNPs on the outcome. The results suggested that by eliminating SNPs related to TG-associated SNPs one by one and recalculating the effects of the remaining SNPs, the overall results remained consistent, suggesting that the pooled effects were not affected (S1 Fig).

These findings support the hypothesis that genetically predicted higher TG levels are associated with an increased risk of EM, while EM itself does not significantly impact TG levels.

### Significant target genes and their source drugs

We analyzed the target genes of 35 fibrates, 12 niacins, and 10 ω-3 PUFA drugs included in DGIDB and ChEMBL.

Out of these, 21 genes showed genetic associations with triglyceride concentration, and among these TG-associated genes, 7 genes showed genetic associations with EM in the present analyses.

Choline Fenofibrate and Ciprofibrate target TNF and SLC9A1 significantly among fibrates, while Fenofibrate targets *APOA1*, *VEGFA*, and *SCN3A*. Among niacin drugs, Probucol significantly targets *ITGAV* and *SCN3A*. *SCN3A* is a significant target for all three ω-3 PUFA drugs. Table 3 displays the information about the included drug targets. Detailed explanations for gene exclusion or inclusion are provided in S1 Table.

**Table 3:** Significant drug targets and their source drugs.

Category	Drugs	Target
Fibrates	Bezafibrate	NA
	Choline Fenofibrate	TNF SLC9A1
	Ciprofibrate	TNF SLC9A1
	Clinofibrate	TNF
	Clofibrate	NA
	Fenofibrate	APOA1 VEGFA SCN3A
Nicotinic Acids	Acipimox	TNF
	Probucol	ITGAV SCN3A
Omega-3 polyunsaturated Fatty Acids	Icosapent Ethyl	SCN3A TNF
	Omega-3-Acid Ethyl Esters	SCN3A
	Omega-3-Carboxylic Acids	BACE1 SCN3A

### Positive control

**Fig 4:**
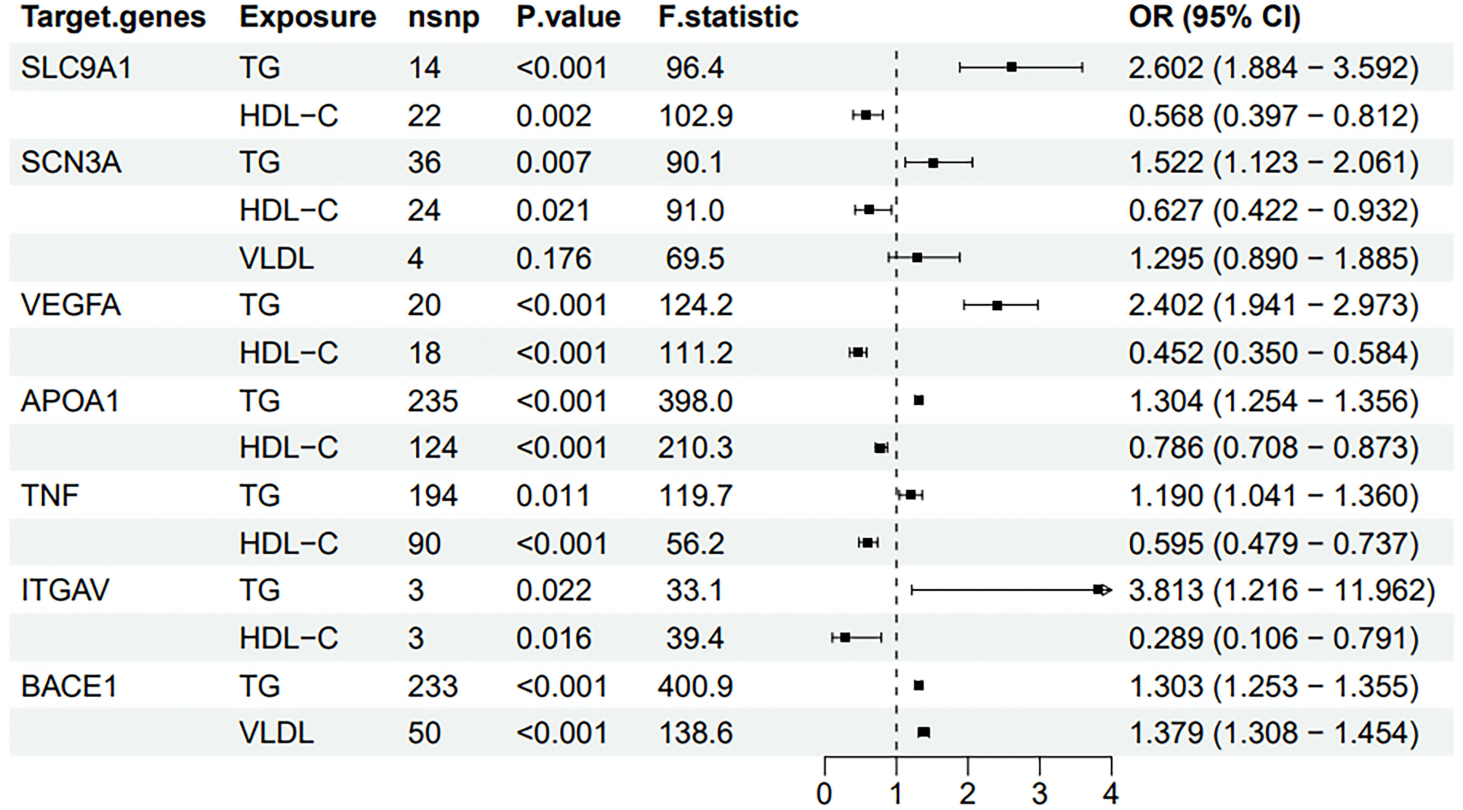
Estimated associations of genetic variation in triglyceride inhibitor targets with positive controls. Exposure: Indicates genes; nsnp: Number of single nucleotide polymorphisms (SNPs) associated with the gene in the sample; *p* value: Significance level, a value less than 0.05 is considered significant; *F* statistic: The *F* statistic, used to assess instrument strength. OR (95% CI): Odds Ratio and its 95% confidence interval. An OR greater than 1 indicates increased risk, while less than 1 indicates reduced risk.

All significant targets associated with TG showed positive associations with CHD risk (OR > 1, *p* < 0.05) (Fig 4).

### Mendelian randomization and sensitivity analysis

**Fig 5:**
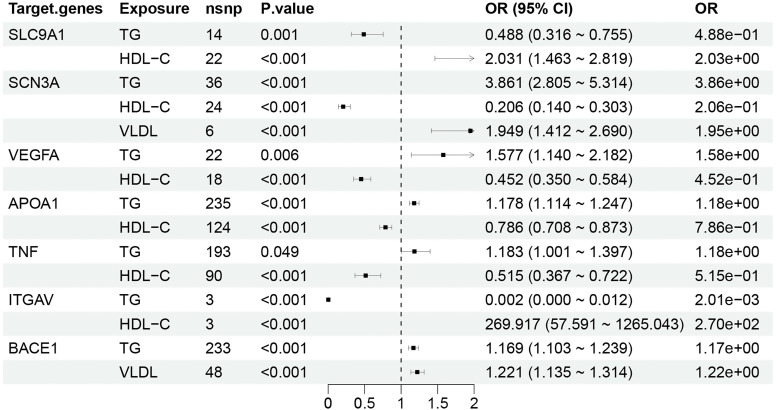
Estimated associations of genetic variation in triglyceride inhibitor targets with EM. Target genes: The genes being analyzed for their association with triglyceride levels. Exposure: The type of lipid measurements includes TG for triglycerides and HDL-C as high-density lipoprotein cholesterol. nsnp: Number of single nucleotide polymorphisms (SNPs) associated with the respective gene. *p* value: Significance level; values below 0.05 were considered statistically significant. *F* statistic: The *F* statistic, used to assess instrument strength. OR (95% CI): Odds Ratio and its 95% confidence interval. An OR greater than 1 indicates increased risk, while less than 1 indicates decreased risk. If the OR is less than 1 and the confidence interval does not include 1, this suggests an association with decreased risk. Narrower confidence interval indicates a more precise estimate.

In the TG-based drug-target MR analysis, genetically proxied effects related to *SCN3A, VEGFA, APOA1, TNF,* and *BACE1* were associated with increased EM risk (OR > 1, *p < 0.05*), whereas genetically proxied effects related to *SLC9A1* and *ITGAV* were associated with reduced EM risk (OR < 1*, p < 0.01).* In sensitivity analysis, *SCN3A* and *BACE1*, which utilized very low density lipoprotein (VLDL) as downstream markers, yielded similar results in TG (OR < 1, *p* < 0.01). However, all included genes exhibited the opposite effect in HDL-C compared to TG and VLDL (OR > 1, *p* < 0.01) (Fig 5).

### Colocalization analysis

The PP.H4 values for all seven genes were below 0.8, indicating limited evidence that TG and EM share a common causal variant within the examined target-gene regions. In contrast, the PP.H3 value for TNF exceeded 0.8, suggesting that TNF may be associated with both traits, but through independent genetic variants. For *APOA1*, *VEGFA*, *SLC9A1*, and *BACE1*, PP.H1 values were higher than the other hypotheses, suggesting stronger evidence for association with TG than with EM in these regions. Overall, the colocalization results did not provide strong support for shared causal variants between TG and EM at the examined drug-target loci (Table 4).

**Table 4:** Colocalization analysis of triglyceride levels and endometriosis in the target gene.

Target gene	nsnps	PP.H0	PP.H1	PP.H2	PP.H3	PP.H4
*APOA1*	11882	7.04 × 10^-314^	5.10 × 10^−01^	6.73 × 10^-314^	4.88 × 10^−01^	1.75 × 10^−03^
*VEGFA*	10265	6.59 × 10^-137^	6.95 × 10^−01^	2.85 × 10^-137^	3.00 × 10^−01^	4.20 × 10^−03^
*SLC9A1*	8894	3.74 × 10^-44^	6.48 × 10^−01^	1.98 × 10^-44^	3.44 × 10^−01^	8.66 × 10^−03^
*SCN3A*	8894	2.80 × 10^-120^	3.91 × 10^−01^	4.29 × 10^-120^	5.98 × 10^−01^	1.07 × 10^−02^
*TNF*	22351	1.08 × 10^-99^	1.55 × 10^−01^	5.82 × 10^-99^	8.38 × 10^−01^	7.51 × 10^−03^
*ITGAV*	9906	1.24 × 10^−04^	7.31 × 10^−02^	4.20 × 10^−04^	2.48 × 10^−01^	6.79 × 10^−01^
*BACE1*	12367	8.59 × 10^-314^	6.23 × 10^−01^	5.18 × 10^-314^	3.75 × 10^−01^	2.14 × 10^−03^

### Transcriptome analysis

Given that fibrates serve as ligands for PPARA, we also examined the association between PPARA and the targets under investigation.

**Fig 6:**
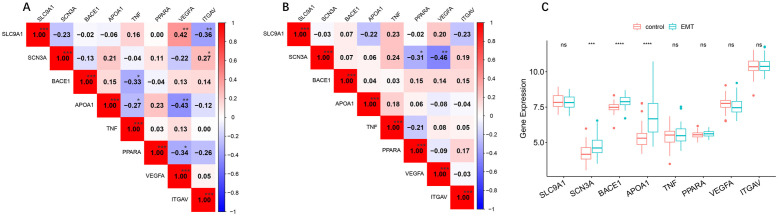
Transcriptome analysis results. (A) Gene-expression correlation matrix of the eight selected genes in EM patients. (B) Gene-expression correlation matrix of the eight selected genes in healthy controls. (C) Box plots comparing the expression levels of the eight selected genes between EM patients and healthy controls. Red indicates a positive correlation, whereas blue indicates a negative correlation. “ns” indicates no statistical significance. Significance: *p* < 0.05 (*), *p <* 0.01 (**), *p <* 0.001 (***), and *p* < 0.0001 (****).

The batch effects were removed from the data. For details, see S2 Fig A and B. Compared with the healthy control group, the EM group exhibited upregulated expression of *SCN3A* (*p* < 0.001), *BACE1*, and *APOA1* (*p* < 0.0001), whereas no statistically significant differences were observed in the expression levels of *SLC9A1*, *TNF*, *PPARA*, *VEGFA*, and *ITGAV* (*p* > 0.05) (Fig 6C).

Gene-expression correlation analysis revealed significant negative correlations between *SCN3A* and *PPARA* (correlation coefficient = −0.31, *p* < 0.05), as well as between *SCN3A* and *VEGFA* (correlation coefficient = −0.46, *p* < 0.01), within the healthy control group (Fig 6B).

In the EM patient group, positive correlations were observed between *SLC9A1* and *VEGFA* (correlation coefficient = 0.42, *p* < 0.01), as well as between *SCN3A* and *ITGAV* (correlation coefficient = 0.27, *p* < 0.05). Conversely, negative correlations were found between *SLC9A1* and *ITGAV* (correlation coefficient = −0.36, *p* < 0.01), *BACE1* and *TNF* (correlation coefficient = −0.33, *p* < 0.05), *APOA1* and *TNF* (correlation coefficient = −0.27, *p* < 0.05), *APOA1* and *VEGFA* (correlation coefficient = −0.43, *p* < 0.01), and *PPARA* and *VEGFA* (correlation coefficient = −0.34, *p* < 0.05) (Fig 6A). The chromosomal locations of the eight genes are shown in S2 Fig C.

### Protein-protein interaction network

**Fig 7:**
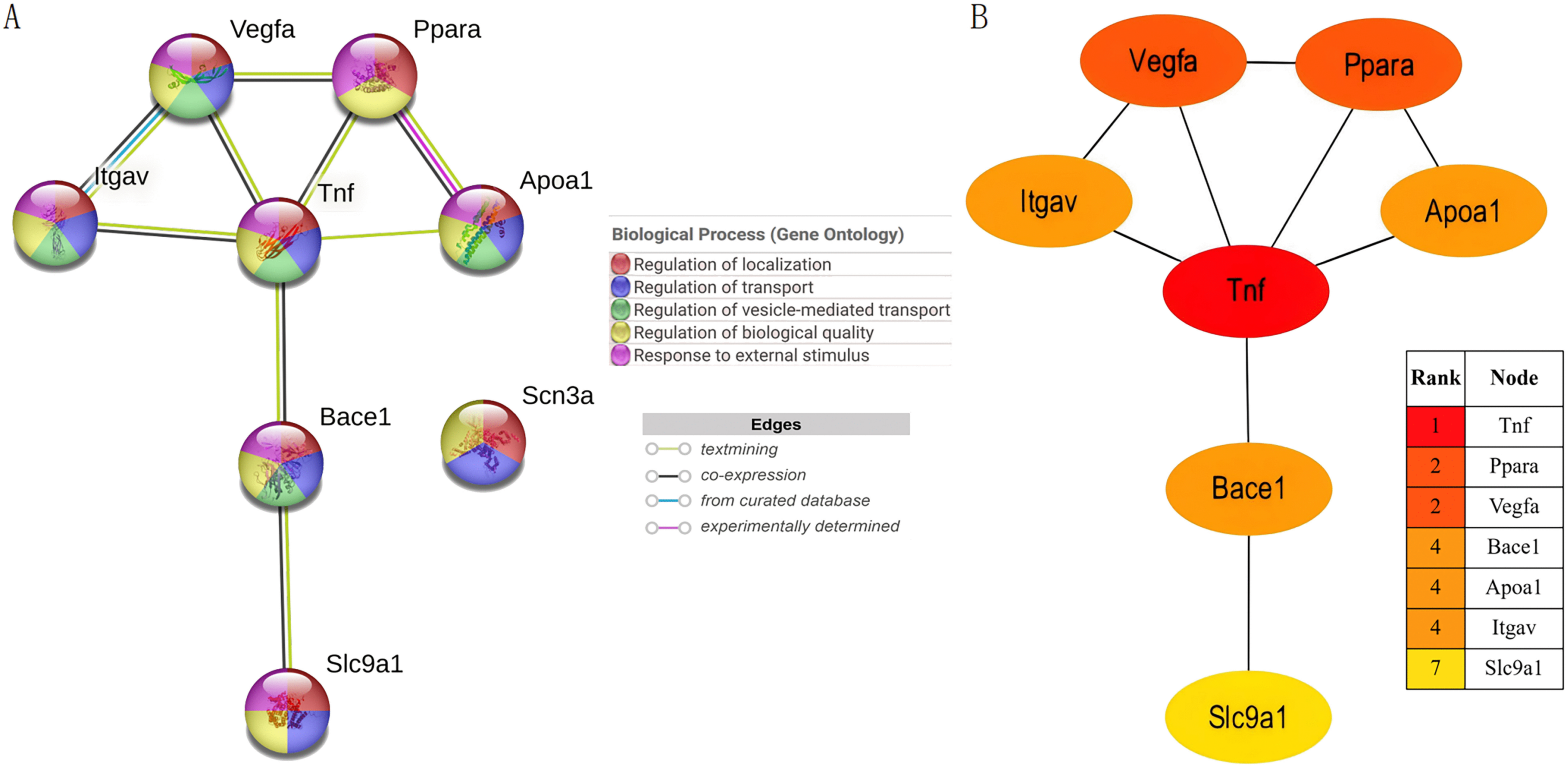
PPI network analysis. (A) PPI network created with STRING. (B) MCC node-importance analysis. In Fig 7A, background colors denote specific biological processes, and different edge styles represent different types of interactions. In Fig 7B, the color coding indicates node importance.

After including *PPARA*, interactions were observed among seven out of the eight genes. *VEGFA, TNF*, and *PPARA* were co-mentioned textual associations (i.e., they were jointly mentioned in the PubMed abstract) as well as co-expression interactions between pairs. *PPARA* and *APOA1* were textually associated, co-expressed, and experimentally confirmed to interact. A textual interaction relationship was indicated between *APOA1* and *TN*F. While *TNF* and *BACE1* exhibited both textual and co-expression interaction relationships. *BACE1* and *SLC9A1* showed both textual and co-expression interaction relationships, as shown in Fig 7A.

The GO enrichment analysis results for the eight genes identified the top five enriched pathways with the smallest FDR values, listed in ascending order, as follows: Regulation of localization, Regulation of transport, Regulation of vesicle-mediated transport, Regulation of biological quality, and Response to external stimulus. All genes were associated with the Regulation of localization pathway. While seven genes (*Tnf, Slc9a1, Apoa1, Ppara, Vegfa, Itgav, Scn3a*) were enriched in the regulation of transport pathway, as depicted in Fig 7A. Gene symbols in the STRING-derived network are shown in the original output format from the STRING database.

### Gene association prediction

Since STRING-based PPI network analysis showed no direct interaction between SCN3A and the other six genes, we utilized the GeneMANIA website to explore indirect relationships between *SCN3A* and the other six genes. Additionally, we employed GeneMANIA to predict potential associated genes that exhibit significant associations with these seven genes.

**Fig 8:**
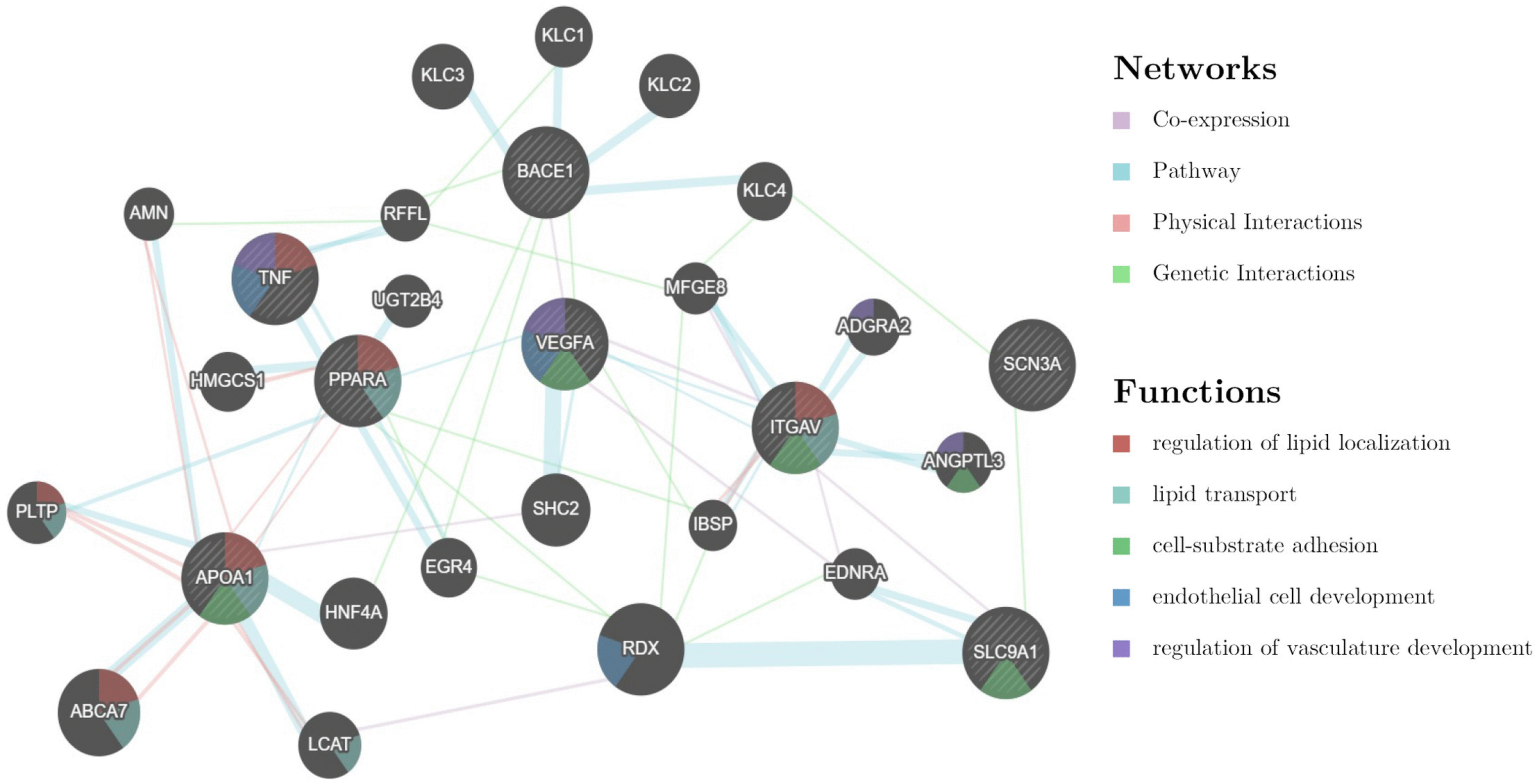
GeneMANIA network to explore the indirect relationships among the seven genes and potential unknown genes that are significantly associated with these seven genes. Each node represents a gene. Lines connecting the nodes signify different types of interactions among the genes. The colors and styles of the edges indicate the nature of these interactions. Larger nodes indicate greater importance and more interactions.

In the regional network, *PPARA*, *TNF*, and *APOA1* exhibited clear physical interaction and pathway relationships. These three genes regulated lipid transport, while *PPARA, APOA1*, and *ITGAV* were involved in lipid transport. *APOA1, VEGFA, ITGAV*, and *SLC9A1* collectively mediated cell-substrate adhesion, *TNF* and *VEGFA* collectively participated in the regulation of vasculature development. *BACE1* exhibited Pathway connections with *KLC1* to *KLC4*, whereas *SCN3A* suggested Genetic Interactions connections with *KLC4* and *SLC9A1* (Fig 8).

### TF regulatory network

**Fig 9:**
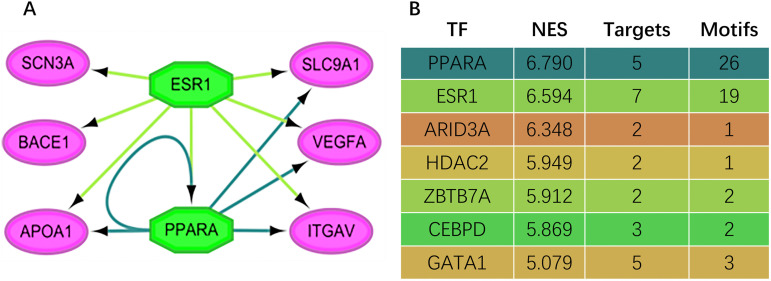
Predicted regulatory network of target genes and TFs. (A) Transcription factor regulatory network. Purple circles indicate target genes regulated by TFs, and green octagons indicate TFs. (B) Transcription factor normalized enrichment score (NES).

The main regulatory factors for the analyzed genes were identified using the Cytoscape plug-in iRegulon, showing that PPARA and estrogen receptor 1 (ESR1) exhibited the highest normalized enrichment score (NES) values among the transcription factors (TFs). PPARA functions as a transcription factor for 5 genes, including itself, while ESR1 acted as a transcription factor for the 7 genes (excluding *TNF)*. The number of motifs associated with PPARA and ESR1 significantly exceeded that of the transcription factor ranked third in terms of NES (Fig 9).

Fig 9A shows the transcription factor regulatory network. Purple circles show target genes regulated by transcription factors. Green octagons indicate transcription factors. Connecting lines indicate regulatory relationships between transcription factors and target genes. The direction of the line indicates the direction of regulation. Fig 9B shows the normalized enrichment score (NES) of transcription factors. The table lists the NES values, the number of target genes and the number of motifs associated with different transcription factors.

## Discussion

### Main findings

This MR study investigated genetic associations between lipid-lowering drug targets and EM risk. The findings suggest that several TG-related drug targets, including targets related to fibrates, niacin, and ω-3 PUFA, may show genetic associations with EM risk. Among these, fibrate-related targets showed relatively broader target coverage in the present analyses. In particular, fenofibrate-related targets were linked to multiple EM-associated signals and showed directionally similar associations in CHD and EM analyses. These findings suggest that fibrate-related pathways may warrant further investigation in EM research.

Both reverse MR and colocalization analysis did not support a bidirectional causal or shared genetic basis between TG levels, TG-lowering drug target genes, and EM. These findings suggest that selected TG-related drug targets may have potential relevance to EM-related biology through TG-associated pathways, although the evidence does not establish a definitive TG-mediated mechanism. This further supports the possibility that TG-related pathways may be linked to EM through multiple targets. The seven identified genes were not selected based on prior assumptions but were derived through a stepwise genetic screening framework integrating TG-related drug targets, genetic associations with TG, and EM-related MR analyses.

Importantly, the colocalization analysis did not provide strong evidence that TG and EM share the same causal variants within the examined drug-target gene regions, as all PP.H4 values were below the commonly used threshold of 0.8. This finding weakens the certainty that the observed drug-target MR associations operate exclusively through shared TG-mediated genetic mechanisms. Therefore, our results should be interpreted as hypothesis-generating genetic evidence suggesting that TG-related pathways and selected lipid-lowering drug targets may be relevant to EM biology, rather than definitive proof of a direct TG-mediated mechanism [56]. This cautious interpretation is also consistent with the broader caution required when interpreting findings from recent endometriosis-related genetic research [57]. Further experimental and clinical studies are needed to validate these candidate targets and clarify their mechanistic roles in EM.

The transcriptome analysis of eight genes revealed that SCN3A, BACE1, and APOA1 exhibited significant up-regulation in EM samples. Moreover, considering the MR results, these three genes showed positive MR-estimated associations with TG, indicated by an OR greater than one. Therefore, in the context of EM, the upregulation of *SCN3A*, *BACE1*, and *APOA1* may reflect TG-related biological dysregulation relevant to EM, although the direction and mechanism of these relationships require experimental validation. These findings suggest that *SCN3A, BACE1,* and *APOA1* may be involved in TG-related biological pathways relevant to EM. However, whether modulation of these genes can alter TG levels or EM-related phenotypes requires further experimental validation. The following is a separate explanation of each target gene.

### TNF

Our study revealed that TNF exhibits the highest importance in the protein interaction network concerning the impact of TG on EM targets. As a cytokine primarily synthesized by activated macrophages and T cells, TNF exerts a pivotal role in the immune response, inflammatory response, and cellular apoptosis. The previous studies observed elevated TNF levels in the peritoneal fluid of women diagnosed with EM, and a positive correlation between TNF levels and the size of endometriotic lesions was identified [58], demonstrating the involvement of TNF in the pathogenesis and progression of EM. As an inflammatory factor, TNF initiated a cascade of inflammatory pathways that can influence the synthesis, breakdown, and transport of lipids [59]. This notion was further supported by the enrichment pathway analysis conducted in our study.

In the context of the inflammatory response, TNF, acting as a pro-inflammatory factor, initiated and intensified the inflammatory response to infections and trauma by attracting additional immune cells and pro-inflammatory mediator signals to the site of injury, thereby exacerbating the development of EM. Furthermore, TNF and interleukin-1 (IL-1) stimulated the expression of cyclooxygenase-2 (COX-2), which, in turn, regulates the synthesis of prostaglandin E2 (PGE2). COX-2 was typically absent under normal conditions and was only upregulated during instances of inflammation and injury [60]. The upregulation of COX-2 expression augments the synthesis of PGE2, which, in turn, can induce the expression of COX-2 and generate surplus PGE2 through a positive feedback loop. The surplus of PGE2 exacerbated inflammatory responses and pain, thereby fostering the progression of EM [61].

Additionally, the enrichment pathway analysis conducted in this study revealed the significant involvement of TNF in angiogenesis. TNF activated the vascular switch in vascular endothelial cells (ECs), thereby facilitating the process of angiogenesis [62]. The formation of new blood vessels facilitated the establishment of endometriotic implants in ectopic locations [63], thereby promoting the occurrence of EM.

### VEGFA

The enriched pathway analysis of the GeneMANIA gene interaction network conducted in this study identified vascular endothelial growth factor A (VEGFA) as another significant contributor to angiogenesis, alongside *TNF*.

VEGFA is recognized as one of the most potent and indispensable angiogenic factors. It stimulates angiogenesis by stimulating the proliferation and migration of vascular endothelial cells (ECs), as well as enhancing vascular permeability [64]. The establishment of an effective blood supply is crucial for the formation, survival, and growth of ectopic endometrial implants, and angiogenesis plays a vital role in this process [65]. Thus, VEGFA may contribute to the development of EM through angiogenesis-related mechanisms. Furthermore, the peritoneal fluid of patients with EM exhibits an elevated presence of activated macrophages [66], which serve as the primary source of VEGFA [67]. This mechanism increases circulating VEGFA concentrations, amplifying its angiogenic effects. Additionally, several studies demonstrate that VEGFA can recruit macrophages [68], thereby establishing a positive feedback loop that results in the substantial accumulation of macrophages. These macrophages can secrete pro-inflammatory cytokines (TNF, IL-1β, and IL-6) [69], further exacerbating the inflammatory environment within the uterine membrane and intensifying the severity of EM [70].

### APOA1

The apolipoprotein A1 gene (*APOA1*) encodes the major component of HDL in plasma. HDL, in turn, delivers APOC2, which activates low-density lipoprotein (LDL) to facilitate TG breakdown [71]. Consequently, *APOA1* is commonly regarded as a protective factor against CHD. Our research indicates that the SNPs within *APOA1* exhibit variations between TG and HDL, resulting in divergent outcomes. Consequently, the protective influence of the *APOA1* gene on CHD primarily stems from its role within HDL. Conversely, within TG, the *APOA1* genetic proxy showed a risk-increasing association with CHD, a pattern that is also observed in EM. In fact, APOA1 serves as a constituent not only of HDL but also of chylomicrons (CM). Given that 85% to 92% of CM consist of TG by mass [72], it is incorrect to employ APOA1 as a sole representation of HDL.

Transcriptome analysis revealed a significant upregulation of *APOA1* in patients with EM, which aligns with the findings reported by JRA Sherwin. According to JRA Sherwin’s research, *APOA1, SFRP4,* and *PAPPA* are typically downregulated by human chorionic gonadotropin (hCG), but in animals with EM, they exhibit upregulation upon hCG stimulation [73]. Similarly, our study found that estrogen receptor 1 (ESR1) functions as a transcription factor for six target genes, including *APOA1*. Given that hCG can suppress the expression of ESR1 [74], the upregulation of *APOA1* in EM is associated with the atypical hormonal effects observed in EM.

### ITGAV

The gene *ITGAV*, which plays a role in the APOA1-mediated signaling pathway [75], is also a target of our study. *ITGAV* encodes the integrin αV subunit, which is a class of membrane protein receptors involved in cell adhesion to the extracellular matrix. Additionally, *ITGAV* has shown to promote adipocyte proliferation [76]. Notably, while high body mass index (BMI) represents a risk factor for coronary heart disease (CHD) [77], it is negatively correlated with the risk of EM [78]. This observation aligns with the findings regarding the performance of ITGAV in TG in CHD and EM, as suggested in our study. EM patients often experience lipodysfunction and fat loss [79]. The dysfunction and reduction of adipocytes, which are the primary cells responsible for storing TG, may lead to an excessive release of TG into the bloodstream. Therefore, the role of ITGAV in both TG and EM can partially explain the phenomenon of elevated TG levels and low BMI in EM patients. This finding also serves as a reminder that in the treatment of EM, it is crucial to reduce triglyceride levels in the blood without compromising the natural formation of adipocytes.

### SCN3A

The *SCN3A* gene encodes Nav1.3, a voltage-gated sodium ion channel protein with selective permeability to sodium ions [80]. Previous studies have suggested that following spinal cord injury, there is abnormal expression of Nav1.3 Na (+) channels in second-order spinal dorsal horn neurons and third-order thalamic neurons along the pain pathway. This change leads to the overexcitability of these neurons, functioning as amplifiers and generators of pain [81]. In the context of EM, it is possible that Nav1.3 channels also contribute to the amplification of pain signals through a similar mechanism. The current study reveals that the expression of *SCN3A* in EM patients was significantly elevated compared to healthy individuals. These findings suggest that SCN3A-related pathways may be involved in pain-related mechanisms in EM; however, whether modulation of SCN3A can influence EM-associated pain requires further experimental investigation.

### SLC9A1

The sodium-hydrogen exchanger-1 (NHE-1), encoded by the SLC9A1 gene, is a plasma membrane protein involved in maintaining intracellular pH and volume [82,83]. NHE-1, along with the sodium-bicarbonate cotransporter NBCn1 (encoded by the *SLC4A7* gene), facilitates the efflux of acid from vascular smooth muscle cells (VSMCs) and ECs in arteries. This acid efflux is essential for maintaining intracellular pH homeostasis [84]. Damage to NHE-1 function disrupts acid excretion, leading to the accumulation of intracellular acid. This accumulation can directly affect the receptors of nociceptive afferent fibers, increasing their sensitivity and resulting in heightened pain perception [85]. Therefore, NHE-1 may represent a potential biological pathway related to EM-associated pain, although its therapeutic relevance requires further validation.

It is important to note that while this study supports the potential of NHE-1 in alleviating EM, activating NHE-1 can promote the proliferation of VSMCs, potentially leading to myocardial hypertrophy [86]. This is particularly concerning for patients with comorbid CHD. Therefore, caution should be exercised when considering drugs that act on NHE-1 in patients with both EM and CHD.

### BACE1

β-site amyloid precursor protein cleaving enzyme 1 (BACE1), also known as β-secretase, is a type 1 membrane protein involved in the cleavage of β-amyloid precursor protein in conjunction with γ-secretase. Its primary function is to produce beta-amyloid. BACE1 has been extensively studied in the context of Alzheimer’s disease (AD). While its expression is highest in the brain, it is also found in various other tissues, including endocrine tissue, pancreas, muscle tissue, respiratory tissue, bone marrow, and lymphoid tissue [87]. In this study, a gene interaction analysis using the GeneMANIA protein interaction network highlighted a connection between TNF and BACE1 through the gene RFFL. RFFL suppresses TNF-induced IKK/NF-κB activation by degrading internalized RIP in endocytic vesicles [88]. NF-κB, in turn, can increase the transcriptional activity of BACE1. Additionally, research has shown that TNF upregulates *BACE1* expression by activating PKC signaling and sequentially cleaving ST6 β-galactoside α-2,6-sialyltransferase (ST6Gal-I) [89], providing an explanation for the observed interconnection between BACE1 and TNF in the PPI network analysis.

Regarding angiogenesis, existing studies have suggested that BACE1 can promote blood vessel formation [90]. In the case of EM, the growth and invasion of endometriotic implants into ectopic sites within the body require the development of new blood vessels [63]. This suggests that BACE1 may be involved in angiogenesis-related processes relevant to EM, which is consistent with the genetic association observed in this study.

Both AD and EM have a higher prevalence in women [91]. In the case of AD, *BACE1* has been a major focus of research as a potential therapeutic target. Another important target associated with AD is *ESR1* [92,93], which is the gene encoding the estrogen receptor alpha. This study suggests that ESR1 serves as a transcription factor for *BACE1*, indicating a regulatory relationship between the two genes. Dysfunction of ESR1 can lead to neuroinflammation, which in turn can result in abnormal gene expression, including the upregulation of *BACE1.* It is important to note that the damaging effects of ESR1 dysfunction are not limited to the brain but can also have an impact on other tissues, including the endometrium, which is geographically distant from the brain. The study implies that the dysregulation of ESR1 in endometrial tissue may contribute to neuroinflammatory processes [93] and abnormal *BACE1* expression, potentially linking the pathogenesis of AD and EM. This suggests that there may be shared molecular mechanisms and pathways between these two conditions, highlighting the importance of considering systemic effects and potential connections between diseases affecting different parts of the body.

### PPARA

Peroxisome proliferator-activated receptors (PPARA, PPAR-β/δ, and PPAR-γ) belong to the nuclear receptor superfamily and are widely recognized for their significant involvement in lipid metabolism. Animal studies have suggested the presence of protein expression of the *PPAR* family, including *PPARA*, in pig endometrial luminal epithelial cells and stromal cells [94]. Despite being an important target of fibrate drugs, this study found no direct evidence linking it to endometriosis (EM) pathogenesis. However, *APOA1, TNF*, and *VEGFA*, which directly interact with *PPARA*, are targets of EM and are affected by fibrate drugs. Simultaneously, PPARA can function as a transcription factor for *SLC9A1, VEGFA, ITGAV*, and *APOA1*. Studies have confirmed that PPARA activation can concomitantly decrease the expression of TNF and VEGFA [95]. As a transcription factor for APOA1, Gervois et al.‘s study suggested that PPARA activation induces the expression of APOA1 and APOA2 in the human liver, leading to an increase in plasma HDL-C and consequent reduction in plasma triglyceride levels [96], thereby potentially influencing EM-related biological processes through modulation of multiple downstream targets.

### Interpretation in the context of what is known

Previous studies have primarily identified the association between TG and EM through clinical investigations. For example, clinical studies have shown that patients with endometriosis (EM) have significantly abnormal lipid profiles, as evidenced by a 38% increase in low-density lipoprotein (LDL), a 30% increase in total cholesterol (TC), and a 27% increase in triglycerides (TG) compared to controls [97]. Patients with endometriosis have an increased risk of hyperlipidemia (OR = 1.25, 95% CI: 1.03–1.52) and a significantly higher risk of hypertension (OR = 1.29, 95% CI:1.07–1.56) [98,99]. The risk of microvascular dysfunction and atherosclerosis was significantly increased in patients with EM due to chronic inflammation and oxidative stress, which together with early menopause (OR = 1.32, 95% CI:1.07–1.64) exacerbated cardiovascular risk [100]. To further comprehend the genetic pathway and the role of TG in EM, the exploration was extended to include the interaction network between TG inhibitor targets. By examining the interactions and relationships among these targets, this study aims to elucidate the molecular mechanisms underlying the influence of TG on EM.

### Strengths and limitations

This study represents the first MR investigation to explore the potential association between lipid-lowering drug-targeted genes and EM risk, offering new insights into the relationship between lipid metabolism and EM. The findings suggest that several TG-related drug targets, including targets related to fibrates, niacin, and ω-3 PUFA, may show genetic associations with EM risk. Among these, fibrate-related targets showed relatively broader target coverage in the present analyses. Furthermore, transcriptome analyses identified *SCN3A*, *BACE1*, and *APOA1* as upregulated genes in EM, providing additional evidence that lipid-related pathways may be relevant to EM biology. However, these findings should be interpreted as hypothesis-generating and require further experimental and clinical validation.

However, this study had several limitations. First, despite the elimination of numerous single nucleotide polymorphisms (SNPs) that might influence the results via phenotypes unrelated to triglyceride (TG) levels, the possibility of horizontal pleiotropy could still introduce bias to the MR findings. Nevertheless, our leave-one-out sensitivity analysis suggested that the association between genetically predicted TG levels and EM risk was not driven by any single SNP. Second, our MR analysis relies on existing genome-wide association study (GWAS) data, which only reflected the lifelong genetic exposure effect. In practical terms, the available data failed to distinguish TG levels at different life stages or within specific time windows. As a result, we were unable to perform sensitivity analyses or subgroup comparisons “across other exposure periods/timings.” This data limitation may have prevented us from uncovering potential variations in effect size related to exposure timing. Third, the study’s focus on the European population limited the generalizability of the findings to other ethnic groups, necessitating further investigation. In terms of genetic association, due to the absence of the VEGFA gene in STRING’s Homo sapiens database, we utilized STRING’s Mus musculus database to construct the PPI network, highlighting the need for further investigation into the applicability of this PPI network in humans. To compensate for this limitation, the study additionally conducted gene association predictions using GeneMANIA for Homo sapiens, providing readers with a point of reference. Fourth, this study was based solely on data from existing databases and lacked experimental validation; therefore, the findings should be interpreted with caution until confirmed by further experimental studies. Nevertheless, our results may offer valuable insights and directions for future experimental research.

Although MR analysis can reduce confounding and reverse causation compared with conventional observational studies, its interpretation relies on several key assumptions, including the validity, independence, and exclusion restriction of genetic instruments [30]. Although multiple sensitivity analyses were performed in this study, residual horizontal pleiotropy or other unmeasured genetic effects cannot be completely excluded [30]. Therefore, the observed genetic associations should be interpreted cautiously and require further experimental and clinical validation. Additional caution is required when interpreting genetic variants located within or near pharmacologically annotated target genes. Such regional variants represent lifelong genetically influenced differences and cannot be regarded as direct equivalents of drug administration, target inhibition or activation, treatment dose, treatment duration, tissue-specific drug action, or off-target effects. Moreover, variants within a target-gene region may influence neighboring genes or regulatory elements, limiting gene-specific pharmacological interpretation. Accordingly, the observed target-region associations should be regarded as exploratory and hypothesis-generating evidence rather than direct estimates of the therapeutic effects of the corresponding drugs [34,35,101].

The transcriptome analyses in this study were based on publicly available datasets generated from different studies, which may involve variations in sample characteristics, experimental platforms, study designs, and experimental conditions. Although batch-effect correction was performed during data processing, residual heterogeneity cannot be completely excluded. Therefore, these transcriptomic findings should be further validated in independent cohorts and experimental studies.

### Implications for clinical practice and future research

Our study provides MR-based evidence suggesting that TG-related pathways and lipid-lowering drug targets may be relevant to EM biology. These findings do not establish triglyceride inhibitors as therapeutic agents for EM, but they highlight fibrate-related pathways as candidates for further mechanistic and translational investigation. Future experimental and clinical studies are needed to determine whether modulation of TG-related pathways can influence EM-related phenotypes.

However, the results also highlight several avenues for future research. Notably, the GWAS data employ in our analysis only provide an estimate of the lifelong exposure effect, without differentiation of TG levels across specific life stages or time windows. Consequently, it was not possible to assess how the associations might differ “across other exposure periods/timings.” Future studies capturing detailed, time-specific exposure information may further elucidate whether the relationship between TG levels and EM risk varies with different exposure periods. Enhancing the granularity of exposure data could contribute to developing more targeted and timely clinical interventions.

In our study, we used MR + multi-omics + drug repurposing strategy to investigate the role of lipid-lowering drugs in endometriosis. MR utilizes genetic variation as an instrumental variable, which can effectively avoid confounding bias and reverse causality in traditional observational studies. Transcriptome analysis, protein interaction networks and pathway enrichment can be used to reveal the “gene-pathway-phenotype” networks of other diseases. Target analysis lays the foundation for repurposing existing drugs and greatly shortens the development cycle of new drugs. Through the integration of genetic association analysis, multi-omics evidence, and drug-target annotation, this study provides a hypothesis-generating framework for investigating lipid-related pathways in complex diseases. Our study may provide a framework for integrating genetic association analysis, multi-omics evidence, and drug-target annotation to generate hypotheses for future translational research. What is more, in the future, we will incorporate electronic prediction of drug-gene interactions (e.g., molecular docking, pathway modeling) into prediction systems. This may improve the predictive value and biological interpretability of future drug-target prioritization studies.

## Conclusion

This study presented MR evidence suggesting that TG-related pathways and triglyceride-lowering drug targets may be relevant to EM biology. Three classes of compounds were identified: fibrates, nicotinic acid, and ω-3 PUFA. Seven candidate drug-target-related genes were implicated: *TNF*, *SLC9A1, APOA1, VEGFA, ITGAV, SCN3A,* and *BACE1*. These selected targets formed an interaction network associated with biological processes potentially relevant to EM, including lipid transport, localization, cell adhesion, and blood vessel development. PPARA and ESR1 were identified as the two TFs with the highest enrichment scores in the regulatory analysis. Considering the genetic evidence and the observed involvement of fibrate-related targets, fenofibrate-related pathways may warrant further mechanistic and translational investigation in EM.

## Supporting information

S1 Fig Leave-one-out sensitivity analysis for TG on EM. (TIF)

S2 Fig Transcriptome analysis results. (A) Analysis of the PCA plot prior to batch effect removal revealed distinct clustering of samples from the same dataset and clear stratification between batches. These findings indicate the presence of substantial batch effects across datasets, which could potentially confound genuine biological variations. (B) Subsequent application of the sva package for batch effect correction resulted in a more uniform distribution of samples across the two-dimensional plane, thereby substantially mitigating the batch effect and enhancing result accuracy. (C) The chromosomal locations of the eight genes.
(TIF)

S1 Table Inclusion or exclusion criteria for genes. (DOCX)

S1 File TG inhibitor target gene information. The compressed archive contains Fibrate target gene information.xlsx, Niacin target gene information.xlsx, and ω-3 PUFA target gene information.
(ZIP)

## References

[1] Horne AW, Missmer SA. Pathophysiology, diagnosis, and management of endometriosis. BMJ. 2022;379:e070750. doi: 10.1136/bmj-2022-070750 36375827

[2] Darbà J, Marsà A. Economic Implications of Endometriosis: A Review. Pharmacoeconomics. 2022;40(12):1143–58. doi: 10.1007/s40273-022-01211-0 36344867

[3] Soliman AM, Surrey E, Bonafede M, Nelson JK, Castelli-Haley J. Real-World Evaluation of Direct and Indirect Economic Burden Among Endometriosis Patients in the United States. Adv Ther. 2018;35(3):408–23. doi: 10.1007/s12325-018-0667-3 29450864 PMC5859693

[4] Duffy JM, Arambage K, Correa FJ, Olive D, Farquhar C, Garry R, et al. Laparoscopic surgery for endometriosis. Cochrane Database of Systematic Reviews. 2014;2014(4):CD011031. doi: 10.1002/14651858.CD011031.pub2 24696265

[5] Chapron C, Marcellin L, Borghese B, Santulli P. Rethinking mechanisms, diagnosis and management of endometriosis. Nat Rev Endocrinol. 2019;15(11):666–82. doi: 10.1038/s41574-019-0245-z 31488888

[6] Nahar K, Khanam NN, Chowdhury AA, Khan NJ, Mohamed Z. Association of Dyslipidemia with Endometriosis: A Case Control Study. Mymensingh Med J. 2023;32(1):118–24. 36594311

[7] Zhang H, Fan Y, Li H, Feng X, Yue D. Genetic association of serum lipids and lipid-modifying targets with endometriosis: Trans-ethnic Mendelian-randomization and mediation analysis. PLoS One. 2024;19(5):e0301752. doi: 10.1371/journal.pone.0301752 38820493 PMC11142702

[8] Zheng R, Du X, Lei Y. Correlations between endometriosis, lipid profile, and estrogen levels. Medicine (Baltimore). 2023;102(29):e34348. doi: 10.1097/MD.0000000000034348 37478235 PMC10662880

[9] Frrhjki J. All about cholesterol: national scientific report. In: Bokeria LA, Oganov RG, editors. Bakulev Scientific Center of Cardiovascular Surgery. Moscow: Russian Academy of Medical Sciences. 2010.

[10] Ahmadian M, Suh JM, Hah N, Liddle C, Atkins AR, Downes M, et al. PPARγ signaling and metabolism: the good, the bad and the future. Nat Med. 2013;19(5):557–66. doi: 10.1038/nm.3159 23652116 PMC3870016

[11] Zandbergen F, Plutzky J. PPARalpha in atherosclerosis and inflammation. Biochim Biophys Acta. 2007;1771(8):972–82. doi: 10.1016/j.bbalip.2007.04.021 17631413 PMC2083576

[12] Miao M, Wang X, Liu T. Targeting PPARs for therapy of atherosclerosis: A review. Int J Biol Macromol. 2023;242(Pt 2):125008. 37217063 10.1016/j.ijbiomac.2023.125008

[13] Jacobson TA. Role of n-3 fatty acids in the treatment of hypertriglyceridemia and cardiovascular disease. Am J Clin Nutr. 2008;87(6):1981S–90S. doi: 10.1093/ajcn/87.6.1981S 18541599

[14] Harris WS, Bulchandani D. Why do omega3 fatty acids lower serum TG?. Curr Opin Lipidol. 2006;17:387–93. 16832161 10.1097/01.mol.0000236363.63840.16

[15] Bays HE, Tighe AP, Sadovsky R, Davidson MH. Prescription omega-3 fatty acids and their lipid effects: physiologic mechanisms of action and clinical implications. Expert Rev Cardiovasc Ther. 2008;6(3):391–409. doi: 10.1586/14779072.6.3.391 18327998

[16] Park Y, Harris WS. Omega-3 fatty acid supplementation accelerates chylomicron triglyceride clearance. J Lipid Res. 2003;44(3):455–63. doi: 10.1194/jlr.M200282-JLR200 12562865

[17] Djuricic I, Calder PC. Beneficial outcomes of omega-6 and omega-3 polyunsaturated fatty acids on human health: an update for 2021. Nutrients. 2021;13(7):2421. doi: 10.3390/nu13072421 34371930 PMC8308533

[18] Feingold KR, Moser A, Shigenaga JK, Grunfeld C. Inflammation stimulates niacin receptor (GPR109A/HCA2) expression in adipose tissue and macrophages. J Lipid Res. 2014;55(12):2501–8. doi: 10.1194/jlr.M050955 25320346 PMC4242443

[19] Ganji SH, Tavintharan S, Zhu D. Lipid Res. 2004;45:1835. 10.1194/jlr.M300403-JLR200 15258194

[20] Sun H, Li G, Zhang W. Niacin activates the PI3K/Akt cascade via PKC- and EGFR-transactivation-dependent pathways through hydroxyl-carboxylic acid receptor 2. PLoS One. 2014;9(11):e112310. doi: 10.1371/journal.pone.0112310 PMC4223033 25375133

[21] Nikas IP, Paschou SA, Ryu HS. The Role of Nicotinamide in Cancer Chemoprevention and Therapy. Biomolecules. 2020;10(3):477. doi: 10.3390/biom10030477 32245130 PMC7175378

[22] Bulun SE, Yildiz S, Adli M, Chakravarti D, Parker JB, Milad M, et al. Endometriosis and adenomyosis: shared pathophysiology. Fertil Steril. 2023;119(5):746–50. doi: 10.1016/j.fertnstert.2023.03.006 36925057

[23] Singh N, Gurav A, Sivaprakasam S, Brady E, Padia R, Shi H, et al. Activation of Gpr109a, receptor for niacin and the commensal metabolite butyrate, suppresses colonic inflammation and carcinogenesis. Immunity. 2014;40(1):128–39. doi: 10.1016/j.immuni.2013.12.007 24412617 PMC4305274

[24] Gasperi V, Sibilano M, Savini I. Niacin in the central nervous system: an update of biological aspects and clinical applications. Int J Mol Sci. 2019;20(4):974. 30813414 10.3390/ijms20040974 PMC6412771

[25] Jiang Y, Jin M, Chen J. Discovery of a novel niacin-lipoic acid dimer N2L attenuating atherosclerosis and dyslipidemia with non-flushing effects. Eur J Pharmacol. 2020;868:172871. 31846627 10.1016/j.ejphar.2019.172871

[26] Jiang S, Li H, Zhang L, Mu W, Zhang Y, Chen T, et al. Generic Diagramming Platform (GDP): a comprehensive database of high-quality biomedical graphics. Nucleic Acids Research. 2024. doi: 10.1093/nar/gkae973 PMC11701665 39470721

[27] Willer CJ, Schmidt EM, Sengupta S, Global Lipids Genetics Consortium. Discovery and refinement of loci associated with lipid levels. Nat Genet. 2013;45(11):1274–83. 24097068 10.1038/ng.2797 PMC3838666

[28] Kurki MI, Karjalainen J, Palta P, Sipilä TP, Kristiansson K, Donner KM, et al. FinnGen provides genetic insights from a well-phenotyped isolated population. Nature. 2023;613(7944):508–18. doi: 10.1038/s41586-022-05473-8 36653562 PMC9849126

[29] Hemani G, Zheng J, Elsworth B, Wade KH, Haberland V, Baird D, et al. The MR-Base platform supports systematic causal inference across the human phenome. Elife. 2018;7:e34408. doi: 10.7554/eLife.34408 29846171 PMC5976434

[30] Bowden J, Davey Smith G, Burgess S. Mendelian randomization with invalid instruments: effect estimation and bias detection through Egger regression. Int J Epidemiol. 2015;44(2):512–25. doi: 10.1093/ije/dyv080 26050253 PMC4469799

[31] Kamat MA, Blackshaw JA, Young R, Surendran P, Burgess S, Danesh J, et al. PhenoScanner V2: an expanded tool for searching human genotype-phenotype associations. Bioinformatics. 2019;35(22):4851–3. doi: 10.1093/bioinformatics/btz469 31233103 PMC6853652

[32] Xu J, Zhang S, Tian Y. Genetic causal association between iron status and osteoarthritis: a two-sample MR. Nutrients. 2022;14(18):3683. 36145059 10.3390/nu14183683 PMC9501024

[33] GTEx Consortium, Laboratory, Data Analysis &Coordinating Center (LDACC)–Analysis Working Group, Statistical Methods groups–Analysis Working Group, Enhancing GTEx (eGTEx) groups, NIH Common Fund, NIH/NCI, et al. Genetic effects on gene expression across human tissues. Nature. 2017;550(7675):204–13. doi: 10.1038/nature24277 29022597 PMC5776756

[34] Luo S, Zheng MH, Wong VWS, Au Yeung SL. Drug-target Mendelian randomisation applied to metabolic dysfunction-associated steatotic liver disease: opportunities and challenges. eGastroenterology. 2024;2(4):e100114. doi: 10.1136/egastro-2024-100114 PMC11770435 39944268

[35] Burgess S, Cronjé HT. Incorporating biological and clinical insights into variant choice for Mendelian randomisation: examples and principles. eGastroenterology. 2024;2(1):e100042. doi: 10.1136/egastro-2023-100042 38362310 PMC7615644

[36] Reiner Ž. Hypertriglyceridaemia and risk of coronary artery disease. Nat Rev Cardiol. 2017;14(7):401–11. doi: 10.1038/nrcardio.2017.31 28300080

[37] Holmes MV, Asselbergs FW, Palmer TM. Mendelian randomization of blood lipids for coronary heart disease. Eur Heart J. 2015;36(9):539–50. doi: 10.1093/eurheartj/ehu001 24474739 PMC4344957

[38] Do R, Willer CJ, Schmidt EM, Sengupta S, Gao C, Peloso GM, et al. Common variants associated with plasma triglycerides and risk for coronary artery disease. Nat Genet. 2013;45(11):1345–52. doi: 10.1038/ng.2795 24097064 PMC3904346

[39] Toutouzas K, Drakopoulou M, Skoumas I, Stefanadis C. Advancing therapy for hypercholesterolemia. Expert Opin Pharmacother. 2010;11(10):1659–72. doi: 10.1517/14656561003774080 20509773

[40] Jain KS, Kathiravan MK, Somani RS, Shishoo CJ. The biology and chemistry of hyperlipidemia. Bioorg Med Chem. 2007;15(14):4674–99. doi: 10.1016/j.bmc.2007.04.031 17521912

[41] Bays H. Clinical overview of Omacor: a concentrated formulation of omega-3 polyunsaturated fatty acids. Am J Cardiol. 2006;98(4A):71i–6i. 10.1016/j.amjcard.2005.12.029 16919519

[42] Edgar R, Domrachev M, Lash AE. Gene Expression Omnibus: NCBI gene expression and hybridization array data repository. Nucleic Acids Res. 2002;30(1):207–10. doi: 10.1093/nar/30.1.207 11752295 PMC99122

[43] Hawkins SM, Creighton CJ, Han DY, et al. Functional microRNA involved in endometriosis. Molecular Endocrinology. 2011;25(5):821–32. doi: 10.1210/me.2010-0450 21436257 PMC3082329

[44] Crispi S, Piccolo MT, D’Avino A, Donizetti A, Viceconte R, Spyrou M, et al. Transcriptional profiling of endometriosis tissues identifies genes related to organogenesis defects. J Cell Physiol. 2013;228(9):1927–34. doi: 10.1002/jcp.24358 23460397

[45] Monsivais D, Dyson MT, Yin P, Coon JS, Navarro A, Feng G, et al. ERβ- and prostaglandin E2-regulated pathways integrate cell proliferation via Ras-like and estrogen-regulated growth inhibitor in endometriosis. Mol Endocrinol. 2014;28(8):1304–15. doi: 10.1210/me.2013-1421 24992181 PMC4116594

[46] Hull ML, Escareno CR, Godsland JM, Doig JR, Johnson CM, Phillips SC, et al. Endometrial-peritoneal interactions during endometriotic lesion establishment. Am J Pathol. 2008;173(3):700–15. doi: 10.2353/ajpath.2008.071128 18688027 PMC2527068

[47] Sha G, Wu D, Zhang L, Chen X, Lei M, Sun H, et al. Differentially expressed genes in human endometrial endothelial cells derived from eutopic endometrium of patients with endometriosis compared with those from patients without endometriosis. Hum Reprod. 2007;22(12):3159–69. doi: 10.1093/humrep/dem266 17956924

[48] Hever A, Roth RB, Hevezi P, Marin ME, Acosta JA, Acosta H, et al. Human endometriosis is associated with plasma cells and overexpression of B lymphocyte stimulator. Proc Natl Acad Sci U S A. 2007;104(30):12451–6. doi: 10.1073/pnas.0703451104 17640886 PMC1941489

[49] Leek JT. svaseq: removing batch effects and other unwanted noise from sequencing data. Nucleic Acids Research. 2014;42(21):e161–e161. doi: 10.1093/nar/gku864 PMC4245966 25294822

[50] Sedgwick P. Spearman’s rank correlation coefficient. BMJ. 2014;349:g7327. 10.1136/bmj.g7327 25432873

[51] Zhang H, Meltzer P, Davis S. RCircos: an R package for Circos 2D track plots. BMC Bioinformatics. 2013;14:244. doi: 10.1186/1471-2105-14-244 23937229 PMC3765848

[52] Yue T, Chen S, Zhu J, Guo S, Huang Z, Wang P, et al. The aging-related risk signature in colorectal cancer. Aging (Albany NY). 2021;13(5):7330–49. doi: 10.18632/aging.202589 33658390 PMC7993742

[53] Szklarczyk D, Gable AL, Lyon D, Junge A, Wyder S, Huerta-Cepas J, et al. STRING v11: protein-protein association networks with increased coverage, supporting functional discovery in genome-wide experimental datasets. Nucleic Acids Res. 2019;47(D1):D607–13. doi: 10.1093/nar/gky1131 30476243 PMC6323986

[54] Franceschini A, Szklarczyk D, Frankild S, Kuhn M, Simonovic M, Roth A, et al. STRING v9.1: protein-protein interaction networks, with increased coverage and integration. Nucleic Acids Res. 2013;41(Database issue):D808–15. doi: 10.1093/nar/gks1094 23203871 PMC3531103

[55] Janky R, Verfaillie A, Imrichová H, Van de Sande B, Standaert L, Christiaens V, et al. iRegulon: from a gene list to a gene regulatory network using large motif and track collections. PLoS Comput Biol. 2014;10(7):e1003731. doi: 10.1371/journal.pcbi.1003731 25058159 PMC4109854

[56] Chen L, Guillot A, Schneider CV. Attention to the misuse of Mendelian randomisation in medical research. eGastroenterology. 2025;3(1):e100187. doi: 10.1136/egastro-2025-100187 40160253 PMC11950933

[57] Shigesi N, Harris HR, Fang H, Ndungu A, Lincoln MR, International Endometriosis Genome Consortium, et al. The phenotypic and genetic association between endometriosis and immunological diseases. Hum Reprod. 2025;40(6):1195–209. doi: 10.1093/humrep/deaf062 40262193 PMC12127507

[58] Harada T, Iwabe T, Terakawa N. Role of cytokines in endometriosis. Fertil Steril. 2001;76(1):1–10. doi: 10.1016/s0015-0282(01)01816-7 11438312

[59] Henning RJ. Obesity and obesity-induced inflammatory disease contribute to atherosclerosis: a review of the pathophysiology and treatment of obesity. Am J Cardiovasc Dis. 2021;11(4):504–29. 34548951 PMC8449192

[60] Lai ZZ, Yang HL, Ha SY. Cyclooxygenase-2 in endometriosis. Int J Biol Sci. 2019;15(13):2783–97. 31853218 10.7150/ijbs.35128 PMC6909960

[61] Ahn SH, Monsanto SP, Miller C, et al. Pathophysiology and immune dysfunction in endometriosis. Biomed Res Int. 2015;2015:795976. 26247027 10.1155/2015/795976 PMC4515278

[62] Rocha ALL, Reis FM, Taylor RN. Angiogenesis and endometriosis. Obstet Gynecol Int. 2013;2013:859619. doi: 10.1155/2013/859619 23766765 PMC3677669

[63] Chung MS, Han SJ. Endometriosis-Associated Angiogenesis and Anti-angiogenic Therapy for Endometriosis. Front Glob Womens Health. 2022;3:856316. doi: 10.3389/fgwh.2022.856316 35449709 PMC9016174

[64] Zhang L, Zhang Y, Li X, Gao H, Chen X, Li P. CircRNA-miRNA-VEGFA: an important pathway to regulate cancer pathogenesis. Front Pharmacol. 2023;14:1049742. doi: 10.3389/fphar.2023.1049742 37234708 PMC10206052

[65] Song WW, Lu H, Hou WJ, Xu GX, Zhang JH, Sheng YH, et al. Expression of vascular endothelial growth factor C and anti-angiogenesis therapy in endometriosis. Int J Clin Exp Pathol. 2014;7(11):7752–9. 25550812 PMC4270624

[66] Shi M, MacLean JA 2nd, Hayashi K. The involvement of peritoneal GATA6+ macrophages in the pathogenesis of endometriosis. Front Immunol. 2024;15:1396000. doi: 10.3389/fimmu.2024.1396000 39192982 PMC11348394

[67] Guo L, Akahori H, Harari E, Smith SL, Polavarapu R, Karmali V, et al. CD163+ macrophages promote angiogenesis and vascular permeability accompanied by inflammation in atherosclerosis. J Clin Invest. 2018;128(3):1106–24. doi: 10.1172/JCI93025 29457790 PMC5824873

[68] Wheeler KC, Jena MK, Pradhan BS, Nayak N, Das S, Hsu C-D, et al. VEGF may contribute to macrophage recruitment and M2 polarization in the decidua. PLoS One. 2018;13(1):e0191040. doi: 10.1371/journal.pone.0191040 29324807 PMC5764356

[69] Jiang I, Yong PJ, Allaire C, Bedaiwy MA. Intricate Connections between the Microbiota and Endometriosis. Int J Mol Sci. 2021;22(11):5644. doi: 10.3390/ijms22115644 34073257 PMC8198999

[70] Santanam N, Murphy AA, Parthasarathy S. Macrophages, oxidation, and endometriosis. Ann N Y Acad Sci. 2002;955(Mar):183–98. 11949947 10.1111/j.1749-6632.2002.tb02779.x

[71] Kwiterovich PO Jr. Diagnosis and management of familial dyslipoproteinemias. Curr Cardiol Rep. 2013;15(6):371. doi: 10.1007/s11886-013-0371-5 23666884

[72] Julve J, Martín-Campos JM, Escolà-Gil JC, et al. Advances in biology, pathology, laboratory testing, and therapeutics. Clin Chim Acta. 2016;455:134–48. doi: 10.1016/j.cca.2016.02.008 26868089

[73] Sherwin JRA, Hastings JM, Jackson KS, Mavrogianis PA, Sharkey AM, Fazleabas AT. The endometrial response to chorionic gonadotropin is blunted in a baboon model of endometriosis. Endocrinology. 2010;151(10):4982–93. doi: 10.1210/en.2010-0275 20668030 PMC2946138

[74] Liao X-H, Wang Y, Wang N, Yan T-B, Xing W-J, Zheng L, et al. Human chorionic gonadotropin decreases human breast cancer cell proliferation and promotes differentiation. IUBMB Life. 2014;66(5):352–60. doi: 10.1002/iub.1269 24753159

[75] Wang D, Liu B, Xiong T, Yu W, She Q. Investigation of the underlying genes and mechanism of familial hypercholesterolemia through bioinformatics analysis. BMC Cardiovasc Disord. 2020;20(1):419. doi: 10.1186/s12872-020-01701-z 32938406 PMC7493348

[76] Morandi EM, Verstappen R, Zwierzina ME. ITGAV and ITGA5 diversely regulate proliferation and adipogenic differentiation of human adipose derived stem cells. Scientific Reports. 2016;6:28889. doi: 10.1038/srep28889 27363302 PMC4929468

[77] Nimptsch K, Konigorski S, Pischon T. Diagnosis of obesity and use of obesity biomarkers in science and clinical medicine. Metabolism. 2019;92:61–70. doi: 10.1016/j.metabol.2018.12.006 30586573

[78] Hong J, Yi KW. What is the link between endometriosis and adiposity? Obstet Gynecol Sci. 2022;65(3):227–33. doi: 10.5468/ogs.21343 35081675 PMC9119730

[79] Dutta M, Anitha M, Smith PB, Chiaro CR, Maan M, Chaudhury K, et al. Metabolomics Reveals Altered Lipid Metabolism in a Mouse Model of Endometriosis. J Proteome Res. 2016;15(8):2626–33. doi: 10.1021/acs.jproteome.6b00197 27246581

[80] Liao S, Liu T, Yang R, Tan W, Gu J, Deng M. Structure and Function of Sodium Channel Nav1.3 in Neurological Disorders. Cell Mol Neurobiol. 2023;43(2):575–84. doi: 10.1007/s10571-022-01211-w 35332400 PMC11415190

[81] Alles SRA, Smith PA. Peripheral Voltage-Gated Cation Channels in Neuropathic Pain and Their Potential as Therapeutic Targets. Front Pain Res (Lausanne). 2021;2:750583. doi: 10.3389/fpain.2021.750583 35295464 PMC8915663

[82] Sarigianni M, Tsapas A, Mikhailidis DP. Na H exchanger-1: a link with atherogenesis? Expert Opinion on Investigational Drugs. 2010;19(12):1545–56. 21047280 10.1517/13543784.2010.532123

[83] Fuster DG, Alexander RT. Traditional and emerging roles for the SLC9 Na+/H+ exchangers. Pflugers Arch. 2014;466(1):61–76. doi: 10.1007/s00424-013-1408-8 24337822

[84] Boedtkjer E, Aalkjaer C. Intracellular pH in the resistance vasculature: regulation and functional implications. J Vasc Res. 2012;49(6):479–96. doi: 10.1159/000341235 22907294

[85] Maddern J, Grundy L, Castro J. Pain in Endometriosis. Front Cell Neurosci. 2020;14:590823. 33132854 10.3389/fncel.2020.590823 PMC7573391

[86] Xia H, Zahra A, Jia M. Na /H Exchanger 1, a Potential Therapeutic Drug Target for Cardiac Hypertrophy and Heart Failure. Pharmaceuticals. 2022;15(7):875. doi: 10.3390/ph15070875 35890170 PMC9318128

[87] Taylor HA, Przemylska L, Clavane EM, Meakin PJ. BACE1: More than just a β-secretase. Obes Rev. 2022;23(7):e13430. doi: 10.1111/obr.13430 35119166 PMC9286785

[88] Liao W, Xiao Q, Tchikov V, Fujita K, Yang W, Wincovitch S, et al. CARP-2 is an endosome-associated ubiquitin ligase for RIP and regulates TNF-induced NF-kappaB activation. Curr Biol. 2008;18(9):641–9. doi: 10.1016/j.cub.2008.04.017 18450452 PMC2587165

[89] Bourne KZ, Ferrari DC, Lange-Dohna C, Rossner S, Wood TG, Perez-Polo JR. Differential regulation of BACE1 promoter activity by nuclear factor-kappaB in neurons and glia upon exposure to beta-amyloid peptides. J Neurosci Res. 2007;85(6):1194–204. doi: 10.1002/jnr.21252 17385716

[90] Paris D, Quadros A, Patel N, DelleDonne A, Humphrey J, Mullan M. Inhibition of angiogenesis and tumor growth by beta and gamma-secretase inhibitors. Eur J Pharmacol. 2005;514(1):1–15. doi: 10.1016/j.ejphar.2005.02.050 15878319

[91] Uddin MS, Rahman MM, Jakaria M, Rahman MS, Hossain MS, Islam A, et al. Estrogen Signaling in Alzheimer’s Disease: Molecular Insights and Therapeutic Targets for Alzheimer’s Dementia. Mol Neurobiol. 2020;57(6):2654–70. doi: 10.1007/s12035-020-01911-8 32297302

[92] Sankaran S, Dubey R, Gomatam A, Chakor R, Kshirsagar A, Lohidasan S. Deciphering the multi-functional role of Indian propolis for the management of Alzheimer’s disease by integrating LC-MS/MS, network pharmacology, molecular docking, and in-vitro studies. Mol Divers. 2024;28(6):4325–42. doi: 10.1007/s11030-024-10818-8 38466554

[93] Liu J, Yuan S, Niu X, Kelleher R, Sheridan H. ESR1 dysfunction triggers neuroinflammation as a critical upstream causative factor of the Alzheimer’s disease process. Aging (Albany NY). 2022;14(21):8595–614. doi: 10.18632/aging.204359 36326669 PMC9699767

[94] Blitek A, Szymanska M. Regulation of expression and role of peroxisome proliferator-activated receptors (PPARs) in luminal epithelial and stromal cells of the porcine endometrium. Theriogenology. 2019;127:88–101. doi: 10.1016/j.theriogenology.2019.01.002 30677596

[95] Clockaerts S, Bastiaansen-Jenniskens YM, Feijt C, De Clerck L, Verhaar JAN, Zuurmond A-M, et al. Cytokine production by infrapatellar fat pad can be stimulated by interleukin 1β and inhibited by peroxisome proliferator activated receptor α agonist. Ann Rheum Dis. 2012;71(6):1012–8. doi: 10.1136/annrheumdis-2011-200688 22307941

[96] Auwerx J, Schoonjans K, Fruchart JC, Staels B. Regulation of triglyceride metabolism by PPARs: fibrates and thiazolidinediones have distinct effects. J Atheroscler Thromb. 1996;3(2):81–9. doi: 10.5551/jat1994.3.81 9226459

[97] Melo AS, Rosa-e-Silva JC, Rosa-e-Silva ACJ de S, Poli-Neto OB, Ferriani RA, Vieira CS. Unfavorable lipid profile in women with endometriosis. Fertil Steril. 2010;93(7):2433–6. doi: 10.1016/j.fertnstert.2009.08.043 19969295

[98] Mu F, Rich-Edwards J, Rimm EB, Spiegelman D, Forman JP, Missmer SA. Association Between Endometriosis and Hypercholesterolemia or Hypertension. Hypertension. 2017;70(1):59–65. doi: 10.1161/HYPERTENSIONAHA.117.09056 28559401 PMC6469492

[99] Cirillo M, Coccia ME, Petraglia F, Fatini C. Role of endometriosis in defining cardiovascular risk: a gender medicine approach for women’s health. Hum Fertil (Camb). 2022;25(4):745–53. doi: 10.1080/14647273.2021.1919764 33926361

[100] Taskin O, Rikhraj K, Tan J, Sedlak T, Rowe TC, Bedaiwy MA. Link between Endometriosis, Atherosclerotic Cardiovascular Disease, and the Health of Women Midlife. J Minim Invasive Gynecol. 2019;26(5):781–4. doi: 10.1016/j.jmig.2019.02.022 31028947

[101] Chen L, Rao Q, Gao M, Lv G, Tacke F. Prospects of Mendelian randomization in hepatology: a comprehensive literature review with practice guidance. Clin Mol Hepatol. 2025;31(4):1115–38. doi: 10.3350/cmh.2025.0541 40485101 PMC12538149

