# Supplementary material for: Genetically predicted triglyceride levels and endometriosis risk: Mendelian randomization with exploratory multi-omics analyses
Source: PLoS One. 2026 Aug 13;21(8):e0356100. doi: 10.1371/journal.pone.0356100 (PMC13472424; doi:10.1371/journal.pone.0356100)
Supplement: Supplementary file 1 [file pone.0356100.s003.docx]

**Supplemental table Inclusion or exclusion criteria for genes**

| **Category** | **gene** | **method** | **NSNPs** | **B** | **SE** | **p-value** | **positive control analysis** | **sensitivity analysis** |
| --- | --- | --- | --- | --- | --- | --- | --- | --- |
| **Fibrates** | ABCA1 | IVW | 12 | -0.393 | 1.096 | 0.511 |  |  |
|  | APOA5 | IVW | 250 | 0.164 | 0.029 | 1.333E-08 |  |  |
|  | APOB | IVW | 71 | 0.236 | 0.080 | 0.003 | F |  |
|  | CFL1 | IVW | 17 | 0.688 | 0.329 | 0.037 | F |  |
|  | CYP2C19 | IVW | 5 | 0.732 | 1.564 | 0.640 |  |  |
|  | CYP2C9 | IVW | 5 | 0.732 | 1.564 | 0.640 |  |  |
|  | HLAA | IVW | 47 | -0.273 | 0.201 | 0.173 |  |  |
|  | KCNH2 | IVW | 8 | 0.456 | 0.394 | 0.248 |  |  |
|  | LPL | IVW | 153 | 0.047 | 0.038 | 0.220 |  |  |
|  | PPARA | IVW | 7 | 1.832 | 0.441 | 3.226E-05 | T | F |
|  | PPARD | IVW | 6 | 2.886 | 0.773 | <0.001 | F |  |
|  | SCARB1 | IVW | 33 | 0.192 | 0.199 | 0.335 |  |  |
|  | SCD | IVW | 2 | -1.430 | 0.993 | 0.150 |  |  |
|  | SLC2A4 | IVW | 17 | -0.999 | 0.328 | 0.002 | F |  |
|  | SLCO1B1 | IVW | 20 | 1.337 | 0.404 | 0.001 | T | F |
|  | TFPI | IVW | 3 | -6.211 | 0.894 | 3.764E-12 | F |  |
|  | TP53 | IVW | 17 | -0.999 | 0.328 | 0.002 | F |  |
|  | VEGFA | IVW | 22 | 0.456 | 0.165 | 0.006 | T | T |
|  | APOA1 | IVW | 250 | 0.164 | 0.029 | 1.33E-08 | T | T |
|  | SLC9A1 | IVW | 14 | -0.717 | 0.222 | 0.001 | T | T |
| **Nicotinic Acids** | BRD1 | IVW | 7 | 2.389 | 0.529 | 6.189E-06 | F |  |
|  | KDM5A | IVW | 2 | 0.403 | 1.525 | 0.791 |  |  |
|  | PIN1 | Wald ratio | 1 | -0.150 | 0.987 | 0.879 |  |  |
|  | PSMB9 | IVW | 133 | 0.198 | 0.100 | 0.047 | F |  |
|  | PTPN11 | IVW | 6 | 0.781 | 0.469 | 0.096 |  |  |
|  | TNF | IVW | 193 | 0.168 | 0.085 | 0.049 | T | T |
|  | TOP1 | IVW | 30 | 0.600 | 0.208 | 0.004 | F |  |
|  | ITGAV | IVW | 3 | -6.211 | 0.894 | 3.764E-12 | T | T |
| **Omega-3 polyunsaturated fatty acids** | BACE1 | IVW | 233 | 0.156 | 0.030 | 1.372E-07 | T | T |
|  | CHRNA4 | IVW | 6 | -0.054 | 0.466 | 0.908 |  |  |
|  | IMPDH2 | IVW | 26 | 0.680 | 0.278 | 0.014 | F |  |
|  | KEAP1 | IVW | 8 | -0.861 | 0.458 | 0.060 |  |  |
|  | PIN1 | IVW | 3 | -1.332 | 0.866 | 0.124 |  |  |
|  | S1PR2 | IVW | 7 | -0.838 | 0.492 | 0.089 |  |  |
|  | SCN3A | IVW | 36 | 1.351 | 0.163 | 1.177E-16 | T | T |
|  | TNF | IVW | 193 | 0.168 | 0.085 | 0.049 | T | T |
|  | TOP1 | IVW | 30 | 0.600 | 0.208 | 0.004 | F |  |
|  | TPSAB1 | IVW | 7 | -0.987 | 0.428 | 0.021 | F |  |

NSNPs: Number of Single Nucleotide Polymorphism; B represents the Beta value, estimating the effect of the exposure variable (drug target) on the outcome variable (endometriosis); SE: Standard Error; IVW: Inverse Variance Weighted;

The table consists of gene targets for all three drugs that significantly influence triglyceride levels. Subsequently, using endometriosis as the outcome, MR analysis was conducted on the targets in the table, with a significance indicated by p-value < 0.05. In the case of significant targets, a positive control analysis was performed, marked as "T," followed by sensitivity analysis; if not marked as "T" in the positive control analysis, sensitivity analysis was not conducted. Only targets marked as "T" in both positive control and sensitivity analyses are identified as drug targets affecting the risk of endometriosis development as triglyceride inhibitors.
